# A tale of two transmitters: serotonin and histamine as in vivo biomarkers of chronic stress in mice

**DOI:** 10.1186/s12974-022-02508-9

**Published:** 2022-06-27

**Authors:** Melinda Hersey, Melissa Reneaux, Shane N. Berger, Sergio Mena, Anna Marie Buchanan, Yangguang Ou, Navid Tavakoli, Lawrence P. Reagan, Claudia Clopath, Parastoo Hashemi

**Affiliations:** 1 Department of Chemistry and Biochemistry, University of South Carolina, Columbia, SC 29208 USA; 2 Department of Pharmacology, Physiology, & Neuroscience, University of South Carolina School of Medicine, Columbia, SC 29209 USA; 3 Department of Bioengineering, Imperial College London, London, SW7 2AZ UK; 4 Columbia VA Health Care Systems, Columbia, SC 29208 USA

**Keywords:** Serotonin, Histamine, Depression, Inflammation, Stress, Biomarkers

## Abstract

**Background:**

Stress-induced mental illnesses (mediated by neuroinflammation) pose one of the world’s most urgent public health challenges. A reliable in vivo chemical biomarker of stress would significantly improve the clinical communities’ diagnostic and therapeutic approaches to illnesses, such as depression.

**Methods:**

Male and female C57BL/6J mice underwent a chronic stress paradigm. We paired innovative in vivo serotonin and histamine voltammetric measurement technologies, behavioral testing, and cutting-edge mathematical methods to correlate chemistry to stress and behavior.

**Results:**

Inflammation-induced increases in hypothalamic histamine were co-measured with decreased in vivo extracellular hippocampal serotonin in mice that underwent a chronic stress paradigm, regardless of behavioral phenotype. In animals with depression phenotypes, correlations were found between serotonin and the extent of behavioral indices of depression. We created a high accuracy algorithm that could predict whether animals had been exposed to stress or not based solely on the serotonin measurement. We next developed a model of serotonin and histamine modulation, which predicted that stress-induced neuroinflammation increases histaminergic activity, serving to inhibit serotonin. Finally, we created a mathematical index of stress, *S*_i_ and predicted that during chronic stress, where *S*_i_ is high, simultaneously increasing serotonin and decreasing histamine is the most effective chemical strategy to restoring serotonin to pre-stress levels. When we pursued this idea pharmacologically, our experiments were nearly identical to the model’s predictions.

**Conclusions:**

This work shines the light on two biomarkers of chronic stress, histamine and serotonin, and implies that both may be important in our future investigations of the pathology and treatment of inflammation-induced depression.

**Supplementary Information:**

The online version contains supplementary material available at 10.1186/s12974-022-02508-9.

## Background

Chronic stress is one of the most urgent public health issues of recent times. Predictions are that in the post-COVID19 era stress-induced mental health illnesses, particularly depression, will be unprecedented [1–3]. The urgency for novel therapeutic treatments, thus, cannot be overstated since existing therapies, including the selective serotonin reuptake inhibitors (SSRIs), do not bring clinical relief to many patients [4]. It continues to be extremely challenging to develop antidepressant therapies, because there are no reliable preclinical screening tests that can accurately predict the outcome of lead compounds. An ideal screening tool would be underpinned by a chemical hypothesis of depression, incorporate measurement of a chemical biomarker of this hypothesis and the response of this biomarker to a candidate agent. A large body of elegant work has supported the original monoamine hypothesis, that surmises that the functionality of monoamines such as serotonin and norepinephrine are impaired during stress and depression [5–9]. The more recent cytokine theory specifies that these impairments are downstream chemical effects of increased immune activity (hence pro-inflammatory cytokines) [10–15]. Both monoamines and pro-inflammatory cytokines have been pharmacologically targeted with some efficacy [4, 16–19], but neither class of analytes are successful, peripheral biomarkers of stress or depression that would facilitate successful diagnosis and/or drug screening [20–24]. In the brain, there has traditionally been a lack of analytical tools to provide temporally, spatially, and chemically relevant measurements to probe theory specific biomarkers.

In this work, we combine niche, in vivo voltammetric serotonin measurement tools and cutting-edge machine learning with mathematical modelling to test the intersection of the monoamine and cytokine theories in a model of chronic stress in mice. We report a significant reduction in extracellular serotonin in the hippocampus in all mice that underwent the chronic stress paradigm, regardless of behavioral phenotype. A machine learning algorithm was able to successfully classify animals as stressed vs. non-stressed based solely on this single serotonin measurement. Excitingly, in animals that displayed depressive-like phenotypes, the magnitude of some behavioral indices of depression correlated well with extracellular serotonin.

The nexus of monoamines and inflammation was next studied via a computational model revealing a bidirectional modulation between serotonin and histamine, a well-known mediator of peripheral inflammation. The model predicted, in agreement with the working hypothesis supported by our current and previous experimental work, that stress-induced inflammation increased histaminergic activity in the brain, a marker of neuroinflammation, serving to inhibit serotonin via H3 receptors. The model revealed the critical importance of histamine in modulating serotonin under stress. We next married the correlative relationships between serotonin and behavior to histamine’s modulation of serotonin to create a mathematical index of stress, which we coin *S*_i_, that serves to stratify the chronically stressed mice. The model predicted that when *S*_i_ was high (high level of stress), this index could be lowered by simultaneously increasing serotonin and decreasing histamine. When pharmacologically performing these experiments suggested by the model, we were able to near-perfectly replicate the model’s predictions and reinstate normal extracellular serotonin.

Our work bridges the two major theories of depression, offering biomarkers for stress and novel pharmacological strategies based on histamine.

## Methods and materials

### Chemicals, reagents, and assays

Calibration solutions were prepared by dissolving serotonin hydrochloride (Sigma-Aldrich Co., St. Louis, MO, USA) in Tris buffer to produce solution concentrations of 10, 25, 50, and 100 nM. Tris buffer consisted of: 15 mM H_2_NC(CH_2_OH)_2_ HCl, 140 mM NaCl, 3.25 mM KCl, 1.2 mM CaCl_2_, 1.25 mM NaH_2_PO_4_.H_2_O, 1.2 mM MgCl_2_, and 2.0 mM Na_2_SO_4_ (Sigma-Aldrich Co., St. Louis, MO, USA) in deionized water and pH adjusted to 7.4. Pharmacological agents were prepared as described below. Escitalopram oxalate (ESCIT) (10 mg kg^−1^, Sigma-Aldrich, St. Louis, MO, USA) and α-fluoromethylhistidine dihyrdrochloride (FMH) (20 mg kg^−1^, Toronto Research Chemicals Inc., Toronto, CAN) were dissolved in saline individually (Hospira, Lake Forest, IL, USA). All pharmaceutical agents were administered via intraperitoneal (*i.p*.) injection at a volume of 5.0 mL kg^−1^ of animal weight. A bioplex was used to analyze cytokines (Bio-Rad Laboratories, Hercules, CA, USA) in plasma at sacrifice.

### Electrode fabrication

Carbon fiber microelectrodes (CFMs) were made as previously described [25]. Briefly, a single carbon fiber (7 μm, Goodfellow, Corporation, Coraopolis, PA, USA) was aspirated into a glass capillary (0.6 mm OD, 0.4 mm ID, 10 cm length; A-M Systems, Sequim, WA, USA) and sealed under gravity and heat by vertical pipette puller (Narishige Group, Tokyo, JAP). Exposed fibers were trimmed to 150 µm under light microscope and silver paint was used to forge an electrical connection to a connection pin. Finally, electrodes were electrodeposited with Nafion™ (LQ-1105, Ion Power Solutions, New Castle DE, USA) as previously described [25].

### Animals

All animal procedures and protocols were performed in accordance with regulations of the Institutional Animal Care and Use Committee (IACUC) at the University of South Carolina, which operates with accreditation from the Association for Assessment and Accreditation of Laboratory Animal Care (AAALAC). Male and female C57BL/6 J mice (Jackson Laboratory, Bar Harbor, ME, USA), arrived at 6–7-week-old group housed, with ad libitum access to food and water, and were kept on a 12 h light/12 h dark cycle (lights off at 7:00 and on at 19:00). A chronic unpredictable mild stress paradigm (CMS) was conducted over a 16-week period and based on previously documented models [26–29]. Two to three mild stressors were performed a day. Stressors included: food or water deprivation, confinement, cage tilt, soiled cage, light during dark cycle, bedding removal, novel object, handling. All stressors were stopped during behavior testing and 12 h leading up to neurochemical studies.

### Behavioral testing

Following the CMS behavioral paradigm, animals underwent previously established behavioral testing for anxiety and depressive-like phenotypes [30]. In a sucrose preference test (SPT), mice were exposed to bottles with water and water with a 1% sucrose solution (Sigma-Aldrich, St. Louis, MO, USA). Water was removed at midnight and then water and water/sucrose was placed at 7:00, water and water/sucrose solutions were weighed before administration, 1 h after placement (8:00), 3 h after placement (11:00), and 12 h after placement (19:00). The test was completed twice, first as a pretest and second as the test. An elevated zero maze (EZM) was conducted as previously described [31]. Each mouse was placed into the closed section of the apparatus (Maze Engineers, Boston, MA, USA) and allowed to explore for 5 min. Time spent in the closed section was measured as an indicator of anxiety-like behavior. A forced swim test (FST) was conducted as described in Yankelevitch-Yahav et al. [32]. In brief, mice were placed in containers filled with water and behavior was observed for 6 min total (the first 2 min were classified as pretest and remaining 4 min were the test). Tail suspension test (TST) was completed as previously described [33]. Each mouse’s tail was attached via tape to the rod and a small plastic, flexible tube was placed on the tail to limit climbing behavior within the apparatus (Maze Engineers, Boston, MA, USA) for the duration of the 6 min test. Percent immobility was measured in the first 2 min (pretest) and the remaining 4 min (test). Rodent behavior was analyzed using Noldus EthoVision (Leesburg, VA, USA).

### Surgical procedures

Surgery was performed following an i.p. injection of 25% w/v urethane (7 μl g^−1^ of body weight) (Sigma-Aldrich Co., dissolved in 0.9% NaCl solution, Hospira, Lake Forest, IL, USA) to maintain and induce anesthesia. Mouse body temperature was maintained using a heating pad (Braintree Scientific, Braintree, MA, USA). Stereotaxic surgery (David Kopf Instruments, Tujunga, CA, USA) was performed, and all coordinates were taken in reference to bregma. A Nafion™-modified CFM was lowered into the CA2 region of the hippocampus (5HT: AP: − 2.91, ML: + 3.35, DV: − 2.5 to − 3.0 mm) or the posterior hypothalamus (HA: AP: − 2.45, ML: + 0.5, DV: − 5.45 to − 5.55 mm) [34] and adjusted until a serotonin/histamine signal was observed. A stimulating electrode (insulated stainless steel, diameter: 0.2 mm, untwisted, Plastics One, Roanoke, VA, USA) was placed into the medial forebrain bundle (5HT: AP: − 1.58, ML: + 1.00, DV: − 4.8 mm) or (HA: AP: − 1.07, ML: + 1.10, DV: − 5.0 mm) [34] and a pseudo Ag/AgCl reference electrode, created by electroplating chloride (30 s in 0.1 M HCl at 5 V) onto a silver wire, was placed into the contralateral hemisphere.

### Voltammetric data collection and analysis

Fast-scan cyclic voltammetry (FSCV) and fast-scan controlled-adsorption voltammetry (FSCAV) were performed using a Dagan potentiostat (Dagan Corporation, Minneapolis, NM, USA), WCCV 3.06 software (Knowmad Technologies LLC, Tucson, AZ, USA), a Pine Research headstage (Pine Research Instrumentation, Durham, NC, USA), and National Instruments DAQ cards NI-6341 and NI-6221 (NI, Austin, TX, USA). For FSCV collection, the “Jackson” serotonin waveform [35] was applied to the electrode at a scan rate of 1000 V s^−1^ and at a frequency of 10 Hz or the histamine waveform, was applied at a scan rate of 600 V s^−1^ and at a frequency of 10 Hz [36]. To evoke serotonin or histamine release, a biphasic stimulation was applied through a linear constant current stimulus isolator (NL800A Neurolog, Medical Systems Corp, Great Neck, NY, USA) with the following parameters: 60 Hz, 350 μA each, 2 ms in width, and 2 s in length. Four control files of serotonin or histamine were collected 10 min apart for each animal prior to injection of pharmacological agents. Following voltammetry data collection, a high voltage was applied to the CFM to lesion the tissue around the electrode which enables electrode placement verification in post-mortem histological analyses.

For basal experiments, control evoked files were collected followed by the methodology being switched to FSCAV. For FSCAV collection, the serotonin waveform was applied at 100 Hz for 2 s, followed by a period of controlled adsorption, where the potential was held at 0.2 V for 10 s; finally, the serotonin waveform was reapplied at 100 Hz, as described in Abdalla et al. [37]. Thirty files (at one file per minute) were collected as control files. Following control files, an *i.p*. injection of saline and then ESCIT (10 mg kg^−1^) *i.p*. was administered and 30 or 60 FSCAV files were collected, respectively, post-treatment. The system was then switched back to traditional FSCV and four post-basal stimulation files were collected. Electrodes were then removed and underwent a post-calibration in solutions of serotonin.

Data were analyzed using WCCV software digital filtration (zero phase, Butterworth, 5 kHz low-pass) and smoothing. For FSCV analysis, the cyclic voltammogram (CV) was used for both histamine and serotonin and the current vs. time (IT) was extracted to resolve release and reuptake of both neurotransmitters. A previously established calibration factor (49.5 ± 10.2 µM/nA) for serotonin analysis [25] and 2.825 µM nA^−1^ for histamine and 11 nM nA^−1^ for serotonin for histamine/serotonin analysis were used to convert current to concentration [36, 38].

For FSCV data, four IT curves were averaged for each animal to establish a control. The average for each individual animal was then combined with the other animals in the group to determine an overall group average. The standard error of the mean (SEM) was calculated using the average IT for each animal (*n* = # animals).

For FSCAV analysis, the third CV after the reapplication of the waveform was selected for quantification, and the peak occurring approximately between 0.4 and 0.85 V was integrated to determine the charge value (pC). Post-calibrations of each electrode, plotting charge (pC) vs. [serotonin] (nM), were used to determine basal concentration.

Animals were excluded if signals were determined to be outliers via the Grubbs test for outliers or if they did not survive the experimental paradigm. To determine the t_1/2_, a code was custom written in excel to fit the reuptake component of the curve and calculate the time taken to reach half of the maximum amplitude. Area under the curve was analyzed via Simpson’s rule with Python.

### Computational model

The bidirectional interaction of histamine and serotonin following chronic stress exposure depression has been modelled (Fig. 3C). The code for the model is written in MATLAB and will be made available on ModelDB.

Here, HA and 5HT denote the histamine and serotonin concentration pool in the hypothalamus, respectively. *τ*_HA_ and *τ*_5HT_ are the decay or reuptake time constants of histamine and serotonin, respectively. *α* is the rate of increase in histamine levels due to serotonin and *β* is rate of decrease in serotonin levels due to histamine. *I*_5HT_ and *I*_HA_ are the tonic supply of serotonin and histamine through other factors.

### Parameter estimation

The parameters of the model have been obtained from the experimental data collected from Samaranayake et al. [38]. The peak responses of histamine and serotonin transients under variation in electrical stimulation amplitude have been used for the steady state analysis and parametrization of the system. The empirical impact of the increase in stimulation amplitude on the physiological dynamics has been modelled through increase in the tonic histamine supply, *I*_HA_.

Furthermore, the model has been extended to predict the impact of stress-induced depression on the serotonergic levels in hippocampus. Since, it is not possible yet to simultaneously measure histamine alongside serotonin in the hippocampus we extrapolated based on findings in the hypothalamus under the assumption that serotonin–histamine dynamics would be similar in these regions. Therefore, based on the established model we computed the concentrations of histamine (Additional file 1: Figure S4). Utilizing the nullcline analysis as before, the parameters have been re-tuned slightly to capture the dynamics well. The magnitude of these parameters is listed in Additional file 1: Table S1. We note that some parameters, including *α*, do vary by brain region when we extrapolated the hypothalamic model to the hippocampus. This is largely influenced by differences in HA and 5HT concentrations by brain region which is then reflected in the generated values.

### Stress index (*S*_i_)

A scalar function, defined as Stress index (*S*_i_), was formulated as a positive-valued function scaled at zero for control and increasing positive values for stress. This function was constructed with the following biological constraints:

1. Histamine is equal to basal brain histamine in controls and higher during stress.
2. Decrease in serotonin from its basal level intensifies the stressed condition caused by increase in histamine. On the other hand, serotonin levels above basal level alleviates stress caused by rise in histamine level.
  $${S}_{\mathrm{i}}= \theta \left(\frac{[\mathrm{HA}]- {[\mathrm{HA}]}_{b}}{{[\mathrm{HA}]}_{b}}\right)\left(\frac{\left[\mathrm{HA}\right]-[{\mathrm{HA}]}_{b}}{{[\mathrm{HA}]}_{b}}\right){\mathrm{e}}^{-\gamma \left( \frac{[5\mathrm{HT}]-{[5\mathrm{HT}]}_{b}}{{[5\mathrm{HT}]}_{b}}\right)}$$

Here, *ϴ* is the Heaviside function, [HA]_*b*_ and [5HT]_*b*_ denote the basal histamine and serotonin concentration, respectively, *γ* is the strength of cooperativity/antagonism by serotonin towards stress, [HA] is the level of histamine and [5HT] is the serotonin level.

### Sensitivity analysis

The model is formulated in the vector form as

$$\frac{\mathrm{d}x}{\mathrm{d}t}= f(x\left(t\right), p)$$

Here, *x* represents the vector of histamine and serotonin concentrations, *f* is the vector function describing the system’s dynamics, and *p* is the parameter of interest under local fluctuation. At the steady state of system:

$$0=f(x\left(p\right), p)$$

Differentiation with respect to *p* gives:

$$0= \frac{\partial f}{\partial x} \frac{\mathrm{d}x}{\mathrm{d}p}+ \frac{\partial f}{\mathrm{d}p}$$

Thus, the absolute sensitivity can be obtained as:

$$\frac{\mathrm{d}x}{\mathrm{d}p}= -{\left[\frac{\partial f}{\partial x}\right]}^{-1} \frac{\partial f}{\partial p}$$

Here, $\left[\frac{\partial f}{\partial x}\right]$ refers to the Jacobian of the vector function. The relative sensitivity $\frac{\mathrm{d}x/x}{\mathrm{d}p/p}$ is obtained by normalizing the absolute sensitivity with respect to the state of the system [39].

### Clustering based on hippocampal serotonin levels

To predict the state of the animal as stressed vs. non-stressed, the extracellular concentrations of hippocampal serotonin in a mice population (non-stress controls and CMS mice) was clustered using k-means clustering technique, with *k* = 2. 80% of the population data was used to train the clustering model in an unsupervised manner and the remaining 20% of the data was used for testing how well the fitted model classified the data. To compute the efficiency of the clustering model, we ran 1000 independent trials with random shuffling of the data and note the frequency of correct classification of the data.

### Statistics

All data are presented as the average with the standard error of the mean (SEM). Significance was determined between two points using a 2-tailed Student’s *t* test and significance was defined and denoted as *p < 0.05.

For FSCV data, the calculated area under the curve of histamine evoked traces was tested to not be drawn from a Gaussian distribution using a Shapiro–Wilk. Analysis was then completed using a Wilcoxon rank sum test (Fig. 3E).

FSCAV data was analyzed using both qualitative and quantitative statistical studies to compare extracellular hippocampal serotonin for the different treatment conditions. Differences in baseline serotonin concentration between control mice and CMS mice (Fig. 7B) were evaluated by analyzing the distribution of the differences between the time series. Additional file 1: Figure S6 A–C shows the histograms of the differences between baseline serotonin concentration for the three groups of mice described in Fig. 7B. Shapiro–Wilk tests were used to test for normality of the distribution of the differences. The test failed in all cases on providing enough evidence to consider the differences to be drawn from a non-Gaussian distribution (*p* = 0.8362, *p* = 0.9458 and *p* = 0.5465, respective to appearance on Additional file 1: Figure S6). The Levene test was used to assess the equality of variance between the three distributions, failing to reject the null hypothesis of equality of variance (*p* = 0.3271). Two-tailed Student’s *t* test assuming equal variance were then used to compare the distributions. Differences in baseline serotonin concentration between control mice and CMS mice were found to be significantly higher than differences between two cohorts of CMS mice (*p* < 0.0001 between distributions shown in Additional file 1: Figure S6A and C, and *p* < 0.0001 between distributions shown in Additional file 1: Figure S6B and C). This confirmed that basal serotonin concentration for CMS mice was lower than control mice prior to any treatment.

Bland–Altman plots were obtained to assess the reproducibility of the signal after saline injection. Additional file 1: Figure S6D–F shows the Bland–Altman plots for the three different acquisitions described in Fig. 7B. No proportional or systematic errors were detected between the initial 30 points (0–30 min) and the following 30 timepoints after saline injection (30–60 min). This confirmed that saline injections did not have any significant impact on the serotonin concentration measurements.

Linear regression analysis was used to model the rate of change of extracellular hippocampal serotonin after treatment (60–120 min) for both control and CMS mice (Fig. 7B). Additional file 1: Figure S7 shows the analysis used to assess the goodness of the fittings. Before any statistical comparison between slopes, the quality of the fittings was assessed by measuring the coefficient of determination and the confidence limits of the models. In all cases, basal serotonin concentration after ESCIT and ESCIT together with FMH administration followed a linear increase over time. Analysis of covariance (ANCOVA) and Tukey post hoc tests were then used to compare the rate of increase of change of serotonin concentration for control and CMS mice (Fig. 7B). Additional file 1: Tables S2 and S3 show the mean estimated slopes for each of the time series included in the ANCOVA test and the results obtained. Additional file 1: Table S4 shows the results of the post hoc pairwise tests. The rate of increase of 5-HT after ESCIT and FMH administration for CMS mice was found to be significantly higher than the rate of increase after ESCIT administration for either control or CMS mice (*p* < 0.0001 in both cases). Equally, the rate of increase of serotonin was also found to be higher after ESCIT administration for control mice compared to CMS mice (*p* < 0.0001). No differences in slope were found between any of the baseline measurements (0–30 min) and after saline injection (30–60 min), which corroborated the lack of a significant impact of saline injection.

A two-way ANOVA (with factors of sex and type of mice) and Tukey–Kramer post hoc analyses were used to reveal significant changes in behavioral test scores and cytokine concentration measurements. A summary of the results is given in Additional file 1: Tables S5 and S6–11. More detailed methods and statistical results can be found in Additional file 1.

## Results

### Serotonin and the monoamine theory in a chronic mild stress paradigm

**Fig. 1:**
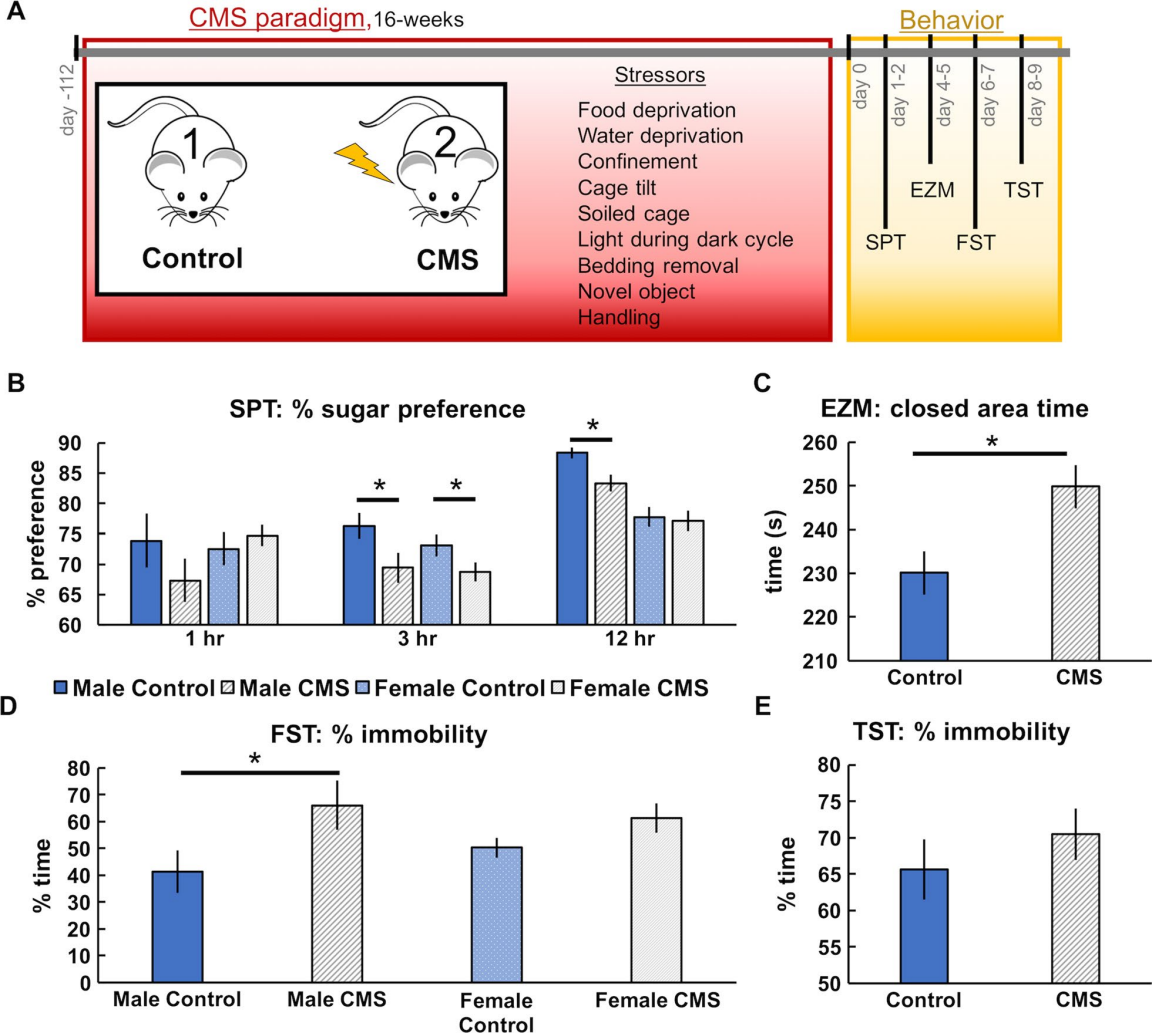
Behavioral changes following CMS. **A** Schematic is shown for the 16-week CMS paradigm and the behavior studies that followed. **B** Average sucrose preference (sucrose water consumed—water consumed/total water consumed) in the SPT for non-stress control (blue; *n* = 40) and CMS (gray; *n* = 39) mice. **C** Average time spent in the closed sections of the EZM is shown for control (blue; *n* = 37) and CMS (gray; *n* = 36) mice (males and females were pooled). **D** Average percentage of time immobile in the FST for male (blue; *n* = 7) and female (light blue; *n* = 8) control mice and male (gray; *n* = 7) and female (light gray; *n* = 6) CMS mice. **E** Average percentage of time immobile in the TST is shown for control (blue; *n* = 37) and CMS (gray; *n* = 36) mice. Statistical significance (*p* < 0.05) is marked by an asterisk

To test serotonin’s roles in the monoamine theory, we utilized the unpredictable chronic mild stress (CMS) paradigm [40]. In a representative cohort of mice that underwent 16 weeks of stressors (Fig. 1A), behavioral analyses for anxiety (EZM) and depression (SPT, TST, and FST) were assessed (Fig. 1B–E). For the SPT (Fig. 1B), significantly less sucrose preference was only found in male mice after 12 h (Control: 88.31 ± 0.92%; CMS: 83.33 ± 1.42%; *p* = 0.0087). For the EZM (1C) CMS-treated mice spent significantly more time in the closed section of the maze than control mice (249.83 ± 2.76 s, 230.09 ± 3.79 s, respectively; *p* < 0.0001) (Fig. 1C). For the FST only male mice showed fewer active behaviors after CMS treatment (Control: 41.36 ± 8.05%; CMS: 66.14 ± 9.13% immobility; *p* = 0.0413) (Fig. 1D). There were no significant differences between control and CMS mice in the TST despite a clear trend (65.64 ± 4.17%, 70.50 ± 3.57%, respectively; *p* = 0.3720) (Fig. 1E). Approximately 74.36% of animals showed anxiety-like phenotypes in the EZM and 41.03% of animals showed depression-like phenotypes after 12 h in the SPT, 76.92% in the FST, and 44.44% in TST. Additional results from behavioral testing can be found in Additional file 1: Table S5.

**Fig. 2:**
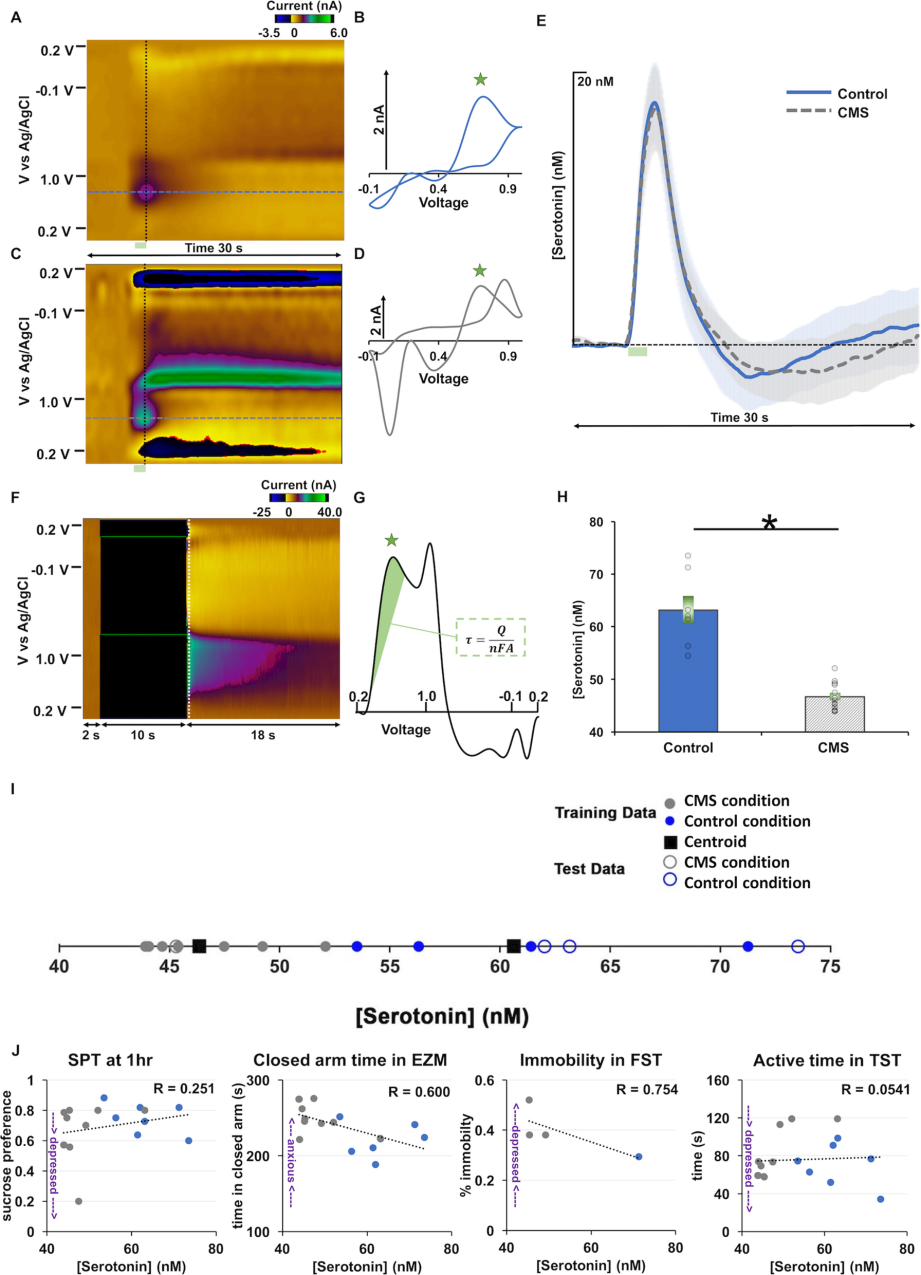
Decreased extracellular serotonin marks chronic stress. **A**, **C** Example color plots from control and CMS mice, respectively. **B**, **D** Example cyclic voltammograms from control and CMS mice, respectively. **E** Evoked hippocampal serotonin concentration with time in control (blue; *n* = 10) and CMS (gray; *n* = 16) mice. **F** Example basal serotonin color plot. **G** Example basal serotonin cyclic voltammogram from which basal serotonin was calculated inset equation (τ = Surface Concentration, Q = Charge, *n* = Charge on the Molecule, F = Faraday Constant, and A = Surface Area). **H** Average basal serotonin in control (blue; *n* = 7) and CMS (gray; *n* = 13) mice are shown as bars and individual animals are denoted by circles. Error is shown as SEM and an asterisk denotes significance via *t* test (*p* < 0.05). **I** *k*-means clustering using extracellular serotonin to predict chronic stress (k = 2). Mice are clustered, based on basal hippocampal serotonin levels, into depressed and control. 80% data is used for training and 20% data is used for testing. **J** Serotonin concentration and sucrose preference at 1 h (*n* = 16), time spent in the closed arms of the EZM (*n* = 17), immobility in the FST (*n* = 4), and active time in the TST (*n* = 17). R = Pearson’s Correlation Coefficient

In the CA2 region of the hippocampus of these same mice, we next measured evoked serotonin with fast-scan cyclic voltammetry (FSCV) and ambient, extracellular serotonin with fast-scan-controlled adsorption voltammetry (FSCAV). There was no difference in evoked serotonin between control and CMS mice (Fig. 2A–E). Using FSCAV we found a significant difference in basal or ambient serotonin in this region (Fig. 2F–H)*,* every single mouse that underwent the chronic stress paradigm had decreased ambient serotonin relative to controls (control: 63.17 ± 0.34 nM, CMS: 46.70 ± 0.26 nM; *t* test, *p* < 0.0001), whether they were behaviorally depressed, or resilient.

Using k-means clustering, a machine learning technique, on the ambient serotonin data we found that the mice are well clustered into two categories: control vs. CMS (Fig. 2I). Via 1000 independent runs of the clustering algorithm with random shuffling of the data, we observed that the clustering almost 90% of the times correctly stratifies the mice.

Next, we plotted indices of behavior vs. basal serotonin concentrations, fitted a linear regression and calculated the Pearson’s correlation coefficient (R value of the fit). In Fig. 2J we present some of the most interesting of those correlations. Blue markers are control mice and gray markers are CMS mice. R values for sucrose preference at 1 h were 0.251, 0.600 for time spent in the closed arms of the EZM, 0.754 for immobility in the FST, and 0.0541 for active time in the TST. Importantly the serotonin concentration in CMS mice cluster towards the high depressive-like behavior end of the scale of all behaviors.

### Inflammation-induced histamine’s modulation of serotonin

**Fig. 3:**
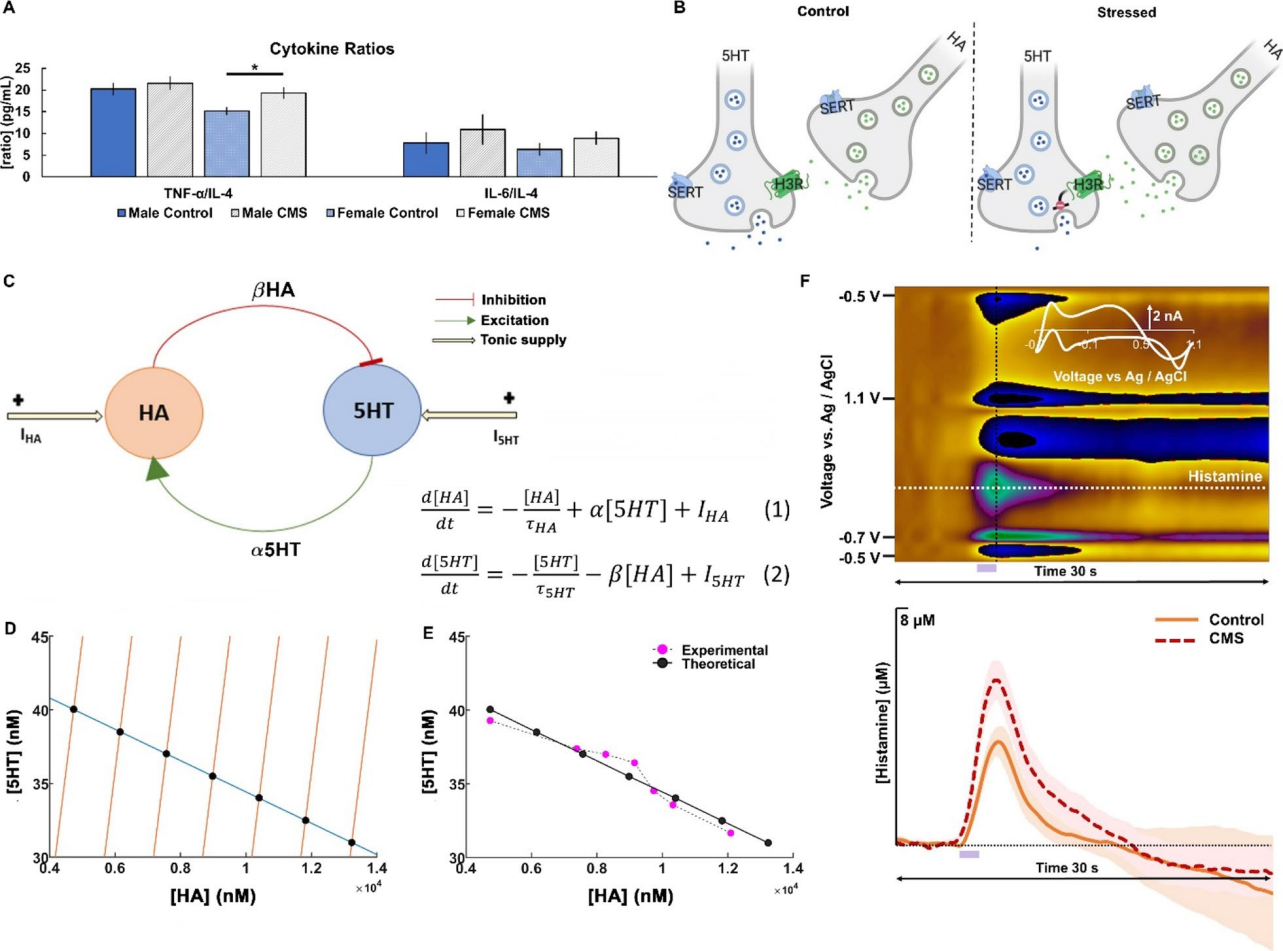
Dynamical interaction between histamine and serotonin with chronic stress. **A** Analysis of cytokine ratios (TNF-α/IL-4 and IL-6/IL4, respectively) for male (blue; *n* = 14) and female (light blue; *n* = 19) control mice and male (gray; *n* = 15) and female (light gray; *n* = 19) CMS mice. Significance was defined as *p* < 0.05 in a *t* test. **B** Schematic showing histaminergic regulation of serotonin via H3 heteroreceptors created with BioRender.com. **C** Schematic diagram and equations depicting the data-based possible interaction between histamine (HA) and serotonin (5HT), involved in stress-induced depression. **D** Nullcline plots of serotonin and histamine are obtained for the basal level of *I*_5HT_ and different levels of *I*_HA_. The intersection points of the curves are the global equilibrium points of the system. **E** Experimental data (solid magenta circles) are obtained from Samaranayake et al. 2016. The theoretical data (solid black circles) have been procured from the nullcline analysis (*r*^2^ = 0.93). **F** Example color plot of histamine FSCV in the hypothalamus. Oxidation of HA can be observed at 0.2 V (green event). Stimulation is marked by a purple box at 5–7 s. Averaged evoked hippocampal HA release for control (orange; *n* = 6) and CMS-treated mice (red; *n* = 5) is shown

To probe how serotonin is modulated in the cytokine theory, we first performed plasma cytokines analysis from the same CMS and control mice (Fig. 3A, same mice as in Figs. 1 and 2). We found no significant difference in peripheral cytokine concentration between CMS and control mice; however, there was significance when the ratios of proinflammatory cytokines to anti-inflammatory cytokines were compared, for example with TNF-a/IL-4 in females (Control: 15.18 ± 0.93 pg mL^−1^; CMS: 19.30 ± 1.30 pg mL^−1^; *p* = 0.0096). CMS-treated mice also had a trend towards an increase in IL-6/IL-4 ratios (*p* = 0.3741 and 0.4497 in male and female mice). We postulate that these markers of inflammation may have only been mildly elevated in the whole blood samples we analyzed and perhaps would have been more remarkable if cytokine measurements had been taken directly from brain tissue.

Next, we turned to histamine for its postulated role in modulating serotonin during stress (Fig. 3B). We constructed a mathematical model based on previously published data, showing a concentration-dependent histaminergic inhibition of serotonin in the posterior hypothalamus (Fig. 3C) [38]. In the model, the change of histamine concentration with time (dH/dt) depends on histamine clearance (− HA/*τ*_HA_), positive modulation of histamine by serotonin (+ α5HT) [41] and constant histamine supply from glia, mast cells, neurons, (*I*_HA_) (Eq. 1). The change of serotonin (d5HT/dt) in turn depends on serotonin clearance (− 5HT/*τ*_5HT_), negative modulation of serotonin by histamine (− *β*HA) [38, 42] and constant ambient serotonin (*I*_5HT_) (Eq. 2). We performed parameterization of the model using nullcline analysis (Fig. 3D). The 5HT-nullcline (blue) consists of the set of points, where serotonin does not change (d5HT/d*t* = 0). The HA-nullclines (orange) are the set of points, where histamine does not change (dHA/d*t* = 0). The rightward orange HA-nullcline lines are for increasing values of *I*_HA_. The black solid markers are the points, where the 5HT- and HA-nullclines intersect and these points are the steady states of the system for different values of *I*_HA_, which means that both histamine and serotonin does not change any longer. We then plotted the steady states generated by the model (black solid markers) superimposed over experimental data, showing excellent agreement (*r*^2^ = 0.93) (Fig. 3F). The model put forth a hypothesis for the decreased serotonin level (as in CMS mice) as a function of elevated histamine level (illustrated by Fig. 3C). We tested this notion experimentally and found in CMS mice a non-significant increase in histamine release in the hypothalamus (Control: 4.28 ± 0.51 µM; CMS: 5.64 ± 0.66 µM; *t* test; *p* = 0.1300) and a significant increase in area under the histamine release curve (Control: 14.51 ± 2.34; CMS: 25.46 ± 2.80; Wilcoxon rank sum test; *p* = 0.0249) (Fig. 3E).

### Histamine clearance predominantly mediates low extracellular serotonin

**Fig. 4:**
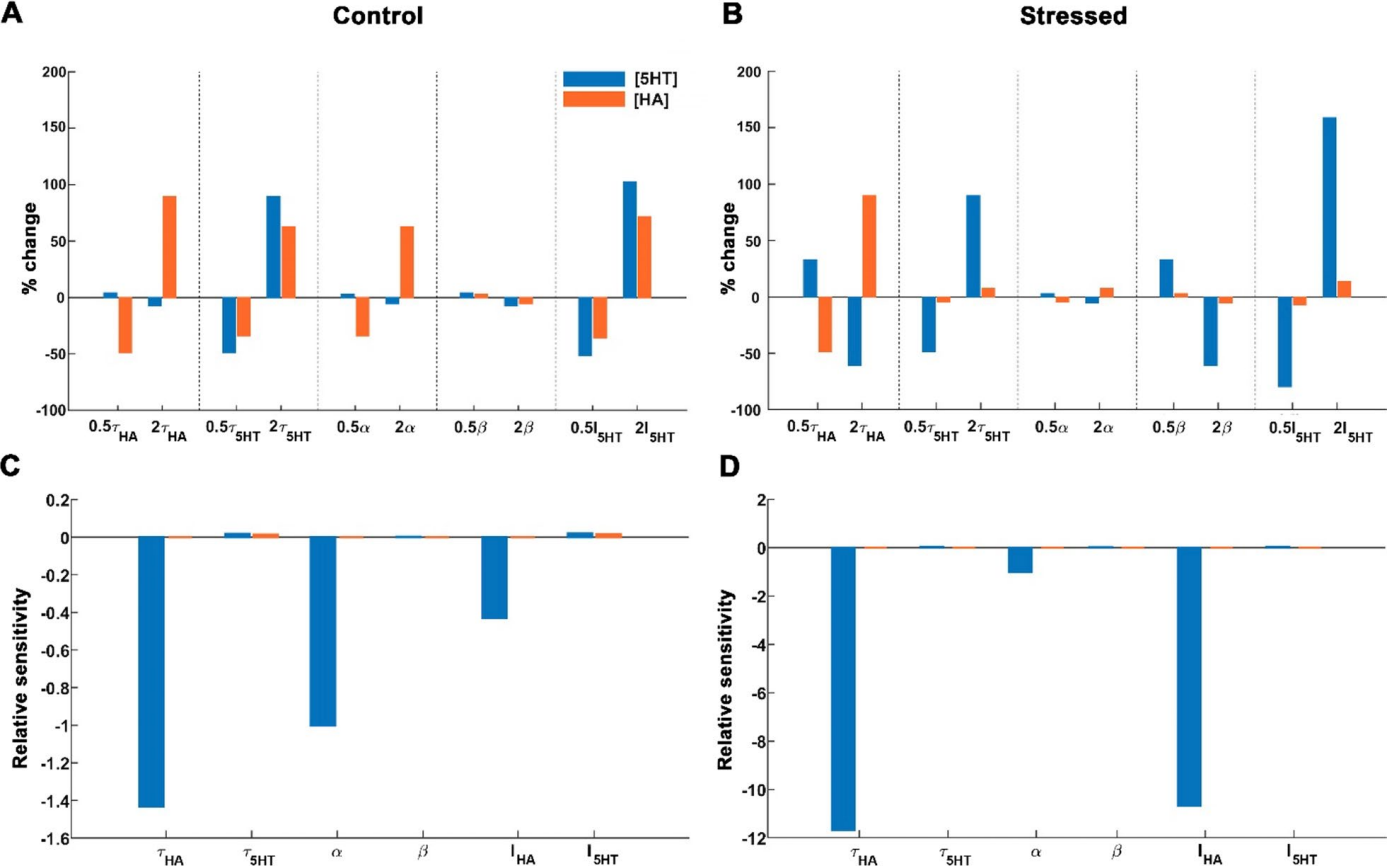
Histamine reuptake is most crucial for regulating stress-induced depression. Two states of the system have been studied **A**–**C** healthy, control condition associated with basal value of *I*_HA_ = 1250 nMs^−1^ and **B**–**D** stressed condition associated with *I*_HA_ = 2*10^4^ nMs^−1^. The serotonin levels are shown in blue bars and the associated histamine levels in orange bars. **A**, **B** Percentage change obtained in the system’s state under parameter variation is shown with respect to the system’s state at basal values of the respective parameters. **C**, **D** Local sensitivity analysis has been performed to identify the parameters towards which the system is most sensitive and vulnerable. Relative sensitivity signifies the vulnerability of a variable towards parameter changes, regardless of the order of the magnitude of the variable as well as the parameter. The basal values of these parameters around which this analysis is performed are *τ*_HA_ = 0.8 s^−1^, *τ*_5HT_ = 0.8 s^−1^, *α* = 69.7961 s^−1^, *β* = 0.0013 s^−1^, *I*_5HT_ = 56.3250 nMs^−1^ and *I*_HA_ = 1250 nMs^−1^ for the healthy condition and *I*_HA_ = 2*10^4^ nMs^−1^ for the stressed condition

We next conducted a global parameter sensitivity analysis on two conditions of the model system, control, healthy state with a basal value of *I*_HA_ (Fig. 4A) and the stress condition with a higher value of *I*_HA_, 20 µMs^−1^ (approximately ten-fold higher than the steady-state ambient histamine) (Fig. 4B) [43]. Parameters were varied independently (on the *x*-axis) from their respective basal value (based on nullcline analysis). The resulting % change in serotonin and histamine from their ambient (*y*-axis). Histamine and serotonin decay rate, *τ*_HA_ and *τ*_5HT_, and the tonic supply of serotonin, *I*_5HT_, greatly impacted the system (largest % change from 0). This was less in the case of serotonin-to-histamine coupling (*α*) and in histamine-to-serotonin coupling (*β*). To uncover how sensitive the system was to a given parameter in control and stressed conditions, we performed a local sensitivity analysis (how small fluctuations affect the system) (Fig. 4C, D). We observed, for both conditions, that serotonin is far more sensitive to small parameter fluctuations than histamine. In this local sensitivity analysis, the most important parameters are the histamine decay rate of (or reuptake rate) *τ*_HA_, the serotonin-to-histamine coupling *α* and tonic supply of histamine *I*_HA_. All three of them, especially the histamine decay rate, have a remarkably strong influence on serotonin. A slight change in these parameters led to a small increase in histamine (orange bars), consistent with our expectations. Strikingly, however, was the effect on serotonin (blue bars) of the change in these parameters of orders of magnitude higher than the effect on histamine.

### ***S***_i_, a novel metric for stratifying stress-induced depression-like behaviors

**Fig. 5:**
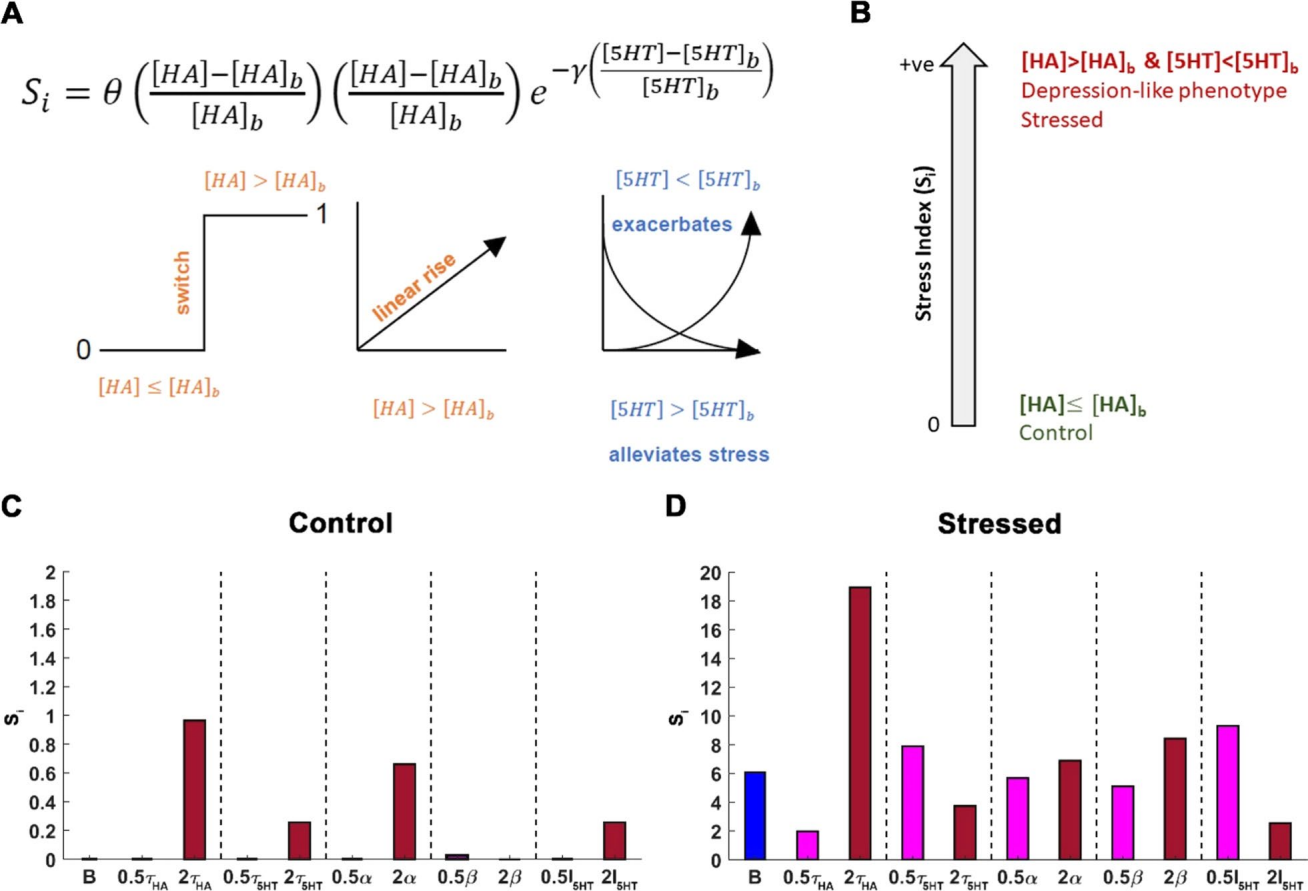
Histamine reuptake critically shapes Stress Index (*S*_i_). **A** This equation describes *S*_i_. The first term is a Heaviside function that acts as a switch filtering the state of the system as either control or stress based on the histamine concentration. A value of zero is ascribed to this term when the histamine concentration equals the basal histamine level. Furthermore, the second term describes the impact histamine levels above the basal histamine value has on *S*_i_ which is a linear rise. The last term describes the contribution of serotonin towards *S*_i_ which either alleviates or exacerbates stress-induced behavior in an exponential manner. **B** Schematic illustration of the metric *S*_i_. *S*_i_ is a positive scalar function that grades stress based on histamine and serotonin steady-state levels. A *S*_i_ value of zero corresponds to controls and an increasing value of *S*_i_ is associated with higher stress. In this model, *S*_i_ associated with the maximum *I*_HA_ = 2*10^4^ nMs^−1^ corresponds to a high level of stress. Furthermore, the normal condition with *I*_HA_ = 1250 nMs^−1^ corresponds to basal or lower histamine levels and the stressed condition is associated with heightened histamine levels and significant reduction in serotonin levels. **C**, **D** Variation in *S*_i_ under a two-fold increase and decrease in the parameter of interest while keeping rest of the parameters fixed. Dark red (magenta) bars refer to a two-fold increase (decrease) in the parameter. The blue bar refers to *S*_i_ for the basal condition i.e. when all the parameters are set to their basal value. **C** The *S*_i_ values for normal conditions are shown. A *S*_i_ of zero exists for basal values of the parameters, shown here in blue bar. It may also be noticed that spanning a parameter range doesn’t cause a significant change in *S*_i_. **D** *S*_i_ values for the stressed condition are also shown. The basal values of the various parameters are *τ*_HA_ = 0.8 s^−1^, *τ*_5HT_ = 0.8 s^−1^, *α *= 69.7961 s^−1^, *β* = 0.0013 s^−1^, *I*_5HT_ = 56.3250 nMs^−1^ and *I*_HA_ = 1250 nMs^−1^ for control and *I*_HA_ = 2*10^4^ nMs^−1^ for stress are obtained by fitting the model to the experimental data. Variation in histamine reuptake rate *τ*_HA_ causes the most significant change in *S*_i_

We next developed a novel general measure, the ‘Stress Index’ (*S*_i_) based on the correlative relationships in Fig. 2 and our serotonin–histamine model. *S*_i_ is a scalar function that correlates stress to chemistry in mice (Fig. 5A), only taking positive values. We utilized the same principles of the hypothalamic co-modulation model because of the well-established presence of inhibitory H3 hetero-receptors on serotonin terminals in the hippocampus [44, 45].

*S*_i_ = 0 refers to control (healthy) behavioral phenotypes and increasing values of *S*_i_ indicate more intense stress conditions. The *S*_i_ index (Eq. 3, Fig. 5A) was constructed with the following biological constraints, based on prior experiments. (1) Histamine concentration (HA) is equal to ambient hypothalamic histamine (HA_b_), (i.e. control models) or higher (i.e. stress models). (2) Decrease in serotonin concentration (5HT), below ambient serotonin concentration (5HT_b_) due to increase in histamine, correlates to behavior (see regressions in Fig. 2J), thus restoring ambient serotonin and histamine back to basal values will decrease *S*_i__:_

3 $${S}_{i}=\Theta \left(\frac{[\mathrm{HA}]-{[\mathrm{HA}]}_{b}}{[{\mathrm{HA}]}_{b}}\right)\left(\frac{[\mathrm{HA}]-{[\mathrm{HA}]}_{b}}{{[\mathrm{HA}]}_{b}}\right){\mathrm{e}}^{-\gamma \left(\frac{[5\mathrm{HT}]-{[5\mathrm{HT}]}_{b}}{[{5\mathrm{HT}]}_{b}}\right)}$$

where *γ* represents the strength of serotonin–histamine cooperation–antagonism (Additional file 1: Figure S3A), [HA]_b_ and [5HT]_b_ represent the basal ambient hypothalamic histamine and serotonin levels, respectively, and [HA] and [5HT] represent the hypothalamic steady-state histamine and serotonin levels. In the model for the hypothalamus the parameters have the values *γ* = 1, [5HT]_b_ = 41.6 nM and [HA]_b_ = 3.32*10^3^ nM.

### Using *S*_i_ to suggest pharmacological approaches

Previously, we studied the parameter sensitivity of serotonin and histamine. Now we use the model to predict the parameter sensitivity of *S*_i_ and, therefore, behavior. We perturbed the model parameters independently and observed that *τ*_HA_, *α* and *I*_5HT_ had great impact on the system (Additional file 1: Figure S4) and on *S*_i_ (Additional file 1: Figure S5). The increase in serotonin is effectuated by an increase in the constant ambient serotonin (*I*_5HT_) or through rapid clearance of histamine, *τ*_HA_ (Additional file 1: Figure S4H). Furthermore, a decrease in *τ*_HA_ decreases *S*_i_. Thus, importantly, the model suggested that an increase in serotonin may be achieved either directly via serotonin or indirectly via a decrease in histamine.

**Fig. 6:**
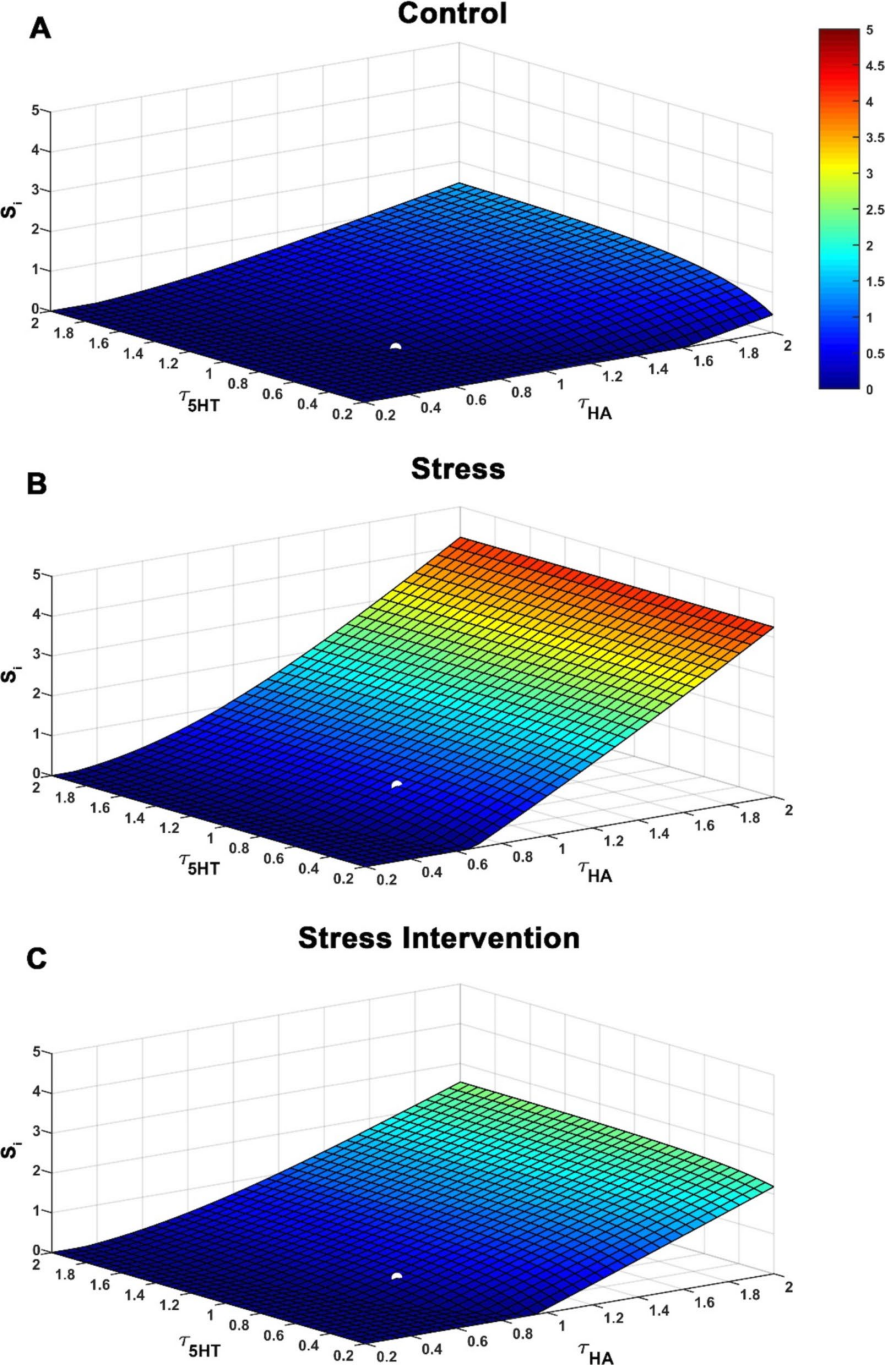
Administration of SSRIs elevates Stress Index. (**A**) SSRIs not only cause an increase in serotonin by blocking serotonin reuptake (the known convention) but also block histamine reuptake leading to a concomitant increase in histamine. This impact is realized in the present model through simultaneous increase in equilibrium histamine and serotonin levels reuptake rates, *τ*_HA_ and *τ*_5HT_, respectively, under (**A**) control, (**B**) stress, (**C**) ESCIT administration conditions. The stress intervention condition is associated with a two-fold decrease in tonic histamine supply, *I*_HA_ which may be achieved through a histamine reducing drug. The white solid circle refers to *S*_i_ for basal parameter values. The graded-color bar denotes the increase in stress index (from blue to red) due to changes in histamine and serotonin concentration. It is observed that blocking histamine leads to a reduced *S*_i_ in **C**. Here, *τ*_HA_ = 0.8 s^−1^, *τ*_5HT_ = 0.8 s^−1^, *α* = 398 s^−1^, *β* = 0.0013 s^−1^, *I*_5HT_ = 118.75 nMs^−1^. *I*_HA_ = 1.25*10^4^ nMs^−1^ is considered for control condition (**A**), *I*_HA_ = 3.88*10^4^ nMs^−1^ for the stressed condition (**B**), and *I*_HA_ = 1.94*10^4^ nMs^−1^ for the stress intervention condition (**C**)

In the context of directly targeting serotonin, the effect of escitalopram (ESCIT), a popular SSRI, was mimicked through a simultaneous decrease in histamine and serotonin reuptake rates (since ESCIT inhibits both serotonin and histamine transport) [46] achieved via increasing *τ*_HA_ and *τ*_5HT_ (Fig. 6A, B). The surface (Fig. 6) illustrates the impact of altering histamine and serotonin reuptake together on *S*_i_. The white solid markers on the surfaces represent *S*_i_ for basal values of *τ*_HA_ and *τ*_5HT_ for control and stress conditions. In both the control and stressed conditions, the model predicts that ESCIT administration will cause an increase in *S*_i_ (via reduction in serotonin, counter to theory), precisely because of ESCIT’s effect on histamine (as described above, small changes in histamine causes large changes in serotonin). The surface for the stress condition has a higher basal level of histamine that allows *S*_i_ to reach four-fold higher. We next probed which conditions would restore *S*_i_ to lower levels and found this achievable by reducing tonic histamine supply, *I*_HA_ (Fig. 6C).

### Pharmacologically targeting histamine and serotonin to restore stress-induced changes in serotonin

**Fig. 7:**
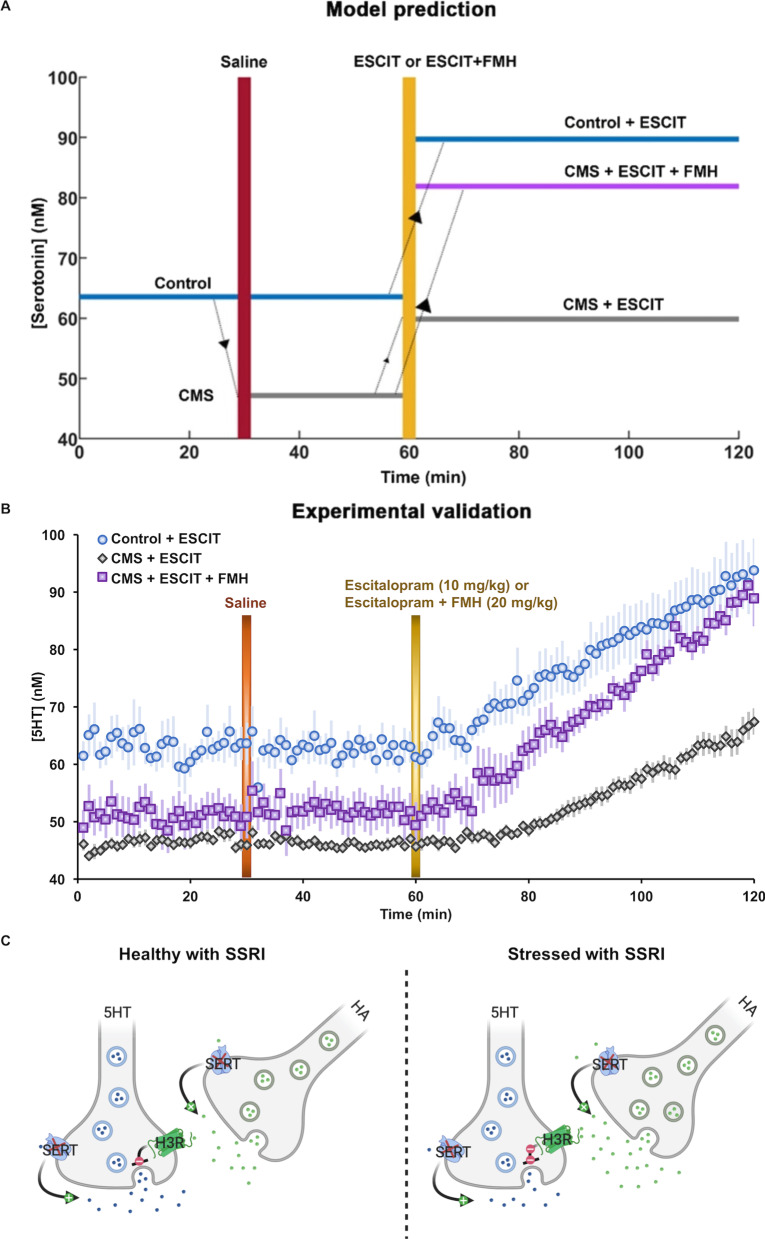
Pharmacologically targeting hippocampal histamine and serotonin concentrations to alleviate stress-induced changes in serotonin. **A** In the model, three representative mice population are considered: control mice administered the SSRI, ESCIT) (in blue), a serotonin elevating drug, chronic mild stress (CMS) mice given ESCIT (in gray) and CMS mice treated with ESCIT and histamine-synthesis blockers α-fluoromethylhistidine (FMH) (in purple). These representative mice population differ from one another based on their serotonin levels and treatment received. It must be noted that our model was studied at steady state. The time plotted here is just to show a more realistic comparison of our model predictions with the experiments conducted to test our observations. In the model ESCIT administration refers to a two-fold increase in *τ*_5HT_ and 1.25-fold increase in *τ*_HA_. Furthermore, FMH administration refers to a two-fold decrease in *I*_HA_. Here, *τ*_HA_ = 0.8 s^−1^, *τ*_5HT_ = 0.8 s^−1^, *α* = 398 s^−1^, *β* = 0.0013 s^−1^, *I*_5HT_ = 118.75 nMs^−1^ and *I*_HA_ = 1.25*10^4^ nMs^−1^ for healthy condition and *I*_HA_ = 3.88*10^4^ nMs^−1^ for the stressed condition. **B** In vivo data showing basal hippocampal serotonin in control mice given saline and then ESCIT (*i.p*., 10 mg kg^−1^, *n* = 7, in blue), CMS-treated mice given saline and then ESCIT (*i.p*., 0.2 mg kg^−1^, *n* = 8, in gray), and CMS-treated mice given saline and then ESCIT (*i.p*., 10 mg kg^−1^) and FMH (*i.p*., 20 mg kg^−1^, *n* = 5, in purple). **C** Modified histamine/serotonin schematic showing the influence of an SSRI on the system in control and chronic stress

Finally, we asked the model how to restore serotonin levels in the stress conditions to healthy levels. We hypothetically considered two representative populations in our model. Control mice were characterized via basal parameter values for the healthy state and the blue line in Fig. 7 shows the basal extracellular serotonin level in this hypothetical control cohort. Stress mice were characterized by increased tonic histamine supply, *I*_HA_ (thus decreased serotonin), represented by the gray line. So far (Fig. 6), the model assumed an equal affinity of ESCIT for inhibiting the serotonin and histamine transporters. ESCIT has much higher affinity for the serotonin transporters than for histamine transporters (the organic cation transporters) [46]. Therefore, we modeled effect of ESCIT as a two-fold increase in the parameter serotonin decay rate *τ*_5HT_ and as a modest increase in the histamine decay rate *τ*_HA_ (1.25τ_HA_), more closely representing the off-target effect of ESCIT on histamine re-uptake. These parameters caused a significant increase in serotonin level in both control and stress conditions. Importantly ESCIT did not restore serotonin levels to pre-stress levels in stress mice. The model hypothesized that these reduced serotonin levels in stress mice could be brought closer to control levels by simultaneously decreasing *I*_HA_ and increasing serotonin with ESCIT (purple line). Figure 7A, thus, shows the model’s hypothesis to our question of how to restore serotonin levels in the stress states to healthy levels. We next tested this hypothesis experimentally. In this experiment, three groups of animal treatments were applied: non-stress control treated with ESCIT (10 mg kg^−1^*, i.p.*), CMS mice treated with saline then ESCIT (10 mg kg^−1^*,* *i.p.*), and CMS mice co-administered ESCIT (10 mg kg^−1^, *i.p.*) and a histamine synthesis inhibitor (FMH, 20 mg kg^−1^, *i.p.*) (Fig. 7B).

Both non-stress controls and CMS-treated mice show increases in ambient serotonin following ESCIT administration (confirmed with a Bland–Altman test); however, post-ESCIT-ambient serotonin is lower in stress mice vs. controls. To simultaneously increase serotonin and decrease histamine, we co-administered ESCIT and a FMH (a suicide inhibitor of histidine decarboxylase) to another cohort of CMS mice. In these mice, we saw that ambient serotonin increased faster and to a level comparable to a control mouse and co-administration of ESCIT and FMH induced robust increases in ambient serotonin (see ANCOVA in supplemental). Strikingly, the experimental data (Fig. 7B) is in very close agreement with the model predictions (Fig. 7A). Thus, dual targeting of serotonin and histamine effectively restores serotonin to control levels in stress mice (see schematic in Fig. 7C).

## Discussion

### Diminished extracellular serotonin marks chronic stress in mice

To identify a biomarker for disease one needs a theory that implicates a chemical change within the pathogenesis of the disease and a way to experimentally validate that chemical change. While this process is straight-forward in many diseases of the periphery (e.g., high blood glucose levels during diabetes), it is an exceptionally challenging task to define biomarkers in the brain due to the complexities of chemically probing this organ [47]. Many decades of elegant work have narrowed down some hypotheses of depression. The long-standing monoamine theory postulates that extracellular levels of the monoamines, particularly serotonin, are lower during depression (inducible by but not limited to chronic stress) [48]. Indeed, SSRIs are still the frontline clinical therapy for depression, the idea being that inhibition of the serotonin transporters (SERTs) will correct the presumed serotonin deficit. However, there has been incongruity in the community about whether brain or body levels of serotonin, or the levels of serotonin metabolites, are reproducibly correlated to depressive state in animals and humans [20]. In addition, while SSRIs do bring clinical relief to many who take them, they are ineffective for the majority. Because of the disagreements about serotonin as a biomarker, the mode of SSRI action and the inability to better screen novel compounds [49, 50], there has been a significant loss of interest in the research community in serotonin-targeting agents.

Serotonin’s roles remain uncertain due to a lack of methods that can capture serotonin signaling in the intact brain on neurotransmission spatial and temporal scales. During the past decade, we have developed a unique set of voltammetric tools that can robustly characterize serotonin neurochemistry in vivo. FSCV can capture evoked serotonin release and reuptake with millisecond time resolution [25] and FSCAV can measure minute-to-minute basal, extracellular concentrations [37]. The hippocampus was targeted with these neurochemical tools because of its well-recognized role of this brain region in the stress and inflammation activated hypothalamic–pituitary–adrenal (HPA) axis [51–53]. Here we apply these tools to bring clarity to the question of serotonin as a biomarker of stress and stress-induced depression-like behaviors in mice. There are many models of behavioral depression in animals and ongoing discussion in the field as to the validity and reproducibility of these tests [28, 54–62]. Largely, there is accord that CMS [40, 63] induces anxiety and depression-like phenotypes in male and female mice [63, 64], and there is evidence for serotonin underpinning these responses [65–71]. It is also clear that stress plays an important role in the chemical pathology of human depression [72]. In a representative cohort of mice that underwent chronic mild stress, we found one or more depression-like behaviors in 41–77% of the mice (depending on the behavior). It is well established that individual differences determine behavioral response to stress including stress resilient subgroups [73–76]. The novel finding here is that *every single mouse* who underwent the CMS paradigm had lower extracellular levels of serotonin compared to controls. In particular, we note that this is consistent between the sexes. In support of this, previous work has shown that there are no significant differences in control and evoked serotonin between male and female mice and no significant difference between how ESCIT increased basal serotonin levels between the sexes [77].

We next asked whether we could mathematically predict, based on this ambient serotonin measurement alone, whether animals had undergone the chronic stress paradigm. Using 1000 independent runs of a clustering algorithm with random shuffling of data, we observe that the clustering almost 90% of the times correctly categorizes the mice population as stressed vs. non-stressed. Thus, we show FSCAV measurements of ambient serotonin level are a stand-alone marker of chronic stress and chronic stress induced depression-like behaviors in mice. Just as significant were the correlations between serotonin concentration and the magnitude of behavioral response, underpinning serotonin’s validity as a behavioral marker. Correlations between voltammetrically measured transmitter levels and behavior have previously been observed [78], in the context of dopamine and learning.

Decreased basal serotonin levels are especially interesting given no change in the evoked serotonin response. We previously described our evoked serotonin response as a function of serotonin synthesis, packaging, release, reuptake, and metabolism [25, 79] so the unchanged signal implies that none of these synaptic parameters changed. This finding clearly shows that there is more to the story than serotonin (and the monoamine hypothesis) alone [19].

### Histamine attenuates extracellular serotonin via stress-induced inflammation

The undeniable comorbidity between stress, inflammation, and depression [10–14] has recently directed the community towards the cytokine theory of depression. The notion here is that the biochemical cascades accompanying immune activity and pro-inflammatory cytokine release in the periphery directly influence the (monoaminergic and/or other) neurochemistry that underlies depression [15]. In this context, we performed plasma cytokine analysis on the (same) CMS and control mice. We found weak relationships between cytokine levels and stress and no relationship between cytokines and behavior (Additional file 1: Figure S8). It is well chronicled that CMS induces chronic inflammation, [80–82]; however, plasma cytokine analysis is not a robust predictor of stress, nor depressive-like behaviors [21, 83–88]. It is likely because collected biochemical samples (quickly metabolized/inactivated) often lose nuance and it is the real-time in vivo signaling that carries the message. However, as with the monoamine theory, it is difficult to measure inflammation signaling in the brain. We recently developed the technology to measure histamine with voltammetry [36, 38]. We showed that measurements of evoked hypothalamic histamine signify acute inflammation [46] and that this inflammation-induced histamine inhibits serotonin release via inhibitory H3 heteroreceptors on serotonin terminals [46]. Many other studies in literature further indicate that histamine strongly influences extracellular serotonin levels [38, 42, 89, 90], thus we decided to focus next on brain histamine.

We first developed a mathematical model of a bidirectional relationship between serotonin and histamine in the hypothalamus accounting for the negative modulation of serotonin by histamine mediated through H3 heteroreceptors (based on our own and others’ previous work) and a positive modulation of histamine by serotonin (based on the *status quo* in the literature). The release of the two neuromodulators was modelled as two independent differential equations. Extracellular histamine and serotonin concentrations were regulated by their neuronal firing rates, vesicular concentrations in the releasing terminals, and reuptake mechanisms at their respective terminals and surrounding glial cells, represented by respective decay time constants. We chose the hypothalamus, because this is the only experimental model available to us to simultaneously test histamine and serotonin dynamics [38].

Nullcline analysis, a common analytical tool used to find the steady state of a system, allowed us to postulate that increasing histamine levels are concurrent with decreasing serotonin, as seen in our CMS mice and also helped to parametrize the model. Next, we voltammetrically measured histamine in the hypothalamus of a second cohort of CMS mice and verified the model’s prediction. Histamine release was elevated in these mice compared to controls. Thus, in addition to serotonin, evoked histamine marks chronic stress. These findings may synergize the monoamine and cytokine theories.

We next dug into the nuances of this intersection and asked which parameter in the model most influenced the model system. We found, using a parameter sensitivity analysis, that changes in histamine were the most significant for their effects on serotonin, specifically, *τ*_HA_, had the biggest impact on the system. This is an exceptionally interesting finding since until recently, active histamine transport had not been described. We and others have found evidence for active histamine reuptake, most likely via the organic cation transporters (OCTs) [46, 91–94]. Even more compelling is recent evidence that agents with antidepressant activity (like SSRIs) have off target effects on histamine reuptake [46].

### *S*_i_ suggests novel approach to improving chemical efficacy of escitalopram via targeted histamine inhibition

Depression in humans is graded using behavioral observations and questionnaires [95] and in animals via an index of a depressive-like behavior [96]. Inaccuracies are innate in both these approaches due to subjectivity (humans) and irreproducibility (animals). By marrying the behavioral and chemical data to our model, we next developed a novel general measure, which we coin ‘the stress index’ (*S*_i_), in the hippocampus. Our model was built on experimental data showing correlations between serotonin and behavior and serotonin–histamine modulation in the hypothalamus. We are not able to measure the modulation of baseline serotonin by histamine in the hippocampus (as in the hypothalamus), because the stimulation releases both serotonin and histamine. Thus we combine the hippocampal ambient serotonin data (from Fig. 2) with our hypothalamic model (Fig. 3) to estimate hippocampal histamine levels and model parameters (Additional file 1: Figure S4A), since inhibitory H3 heteroreceptors are also found on serotonin terminals in the hippocampus [44, 45].

To make sure that *S*_i_ is robust against the exact equation used, we considered many different functional dependencies for histamine and serotonin on *S*_i_ (Additional file 1: Figure S2). Remarkably, the system shows conserved behavior for all the different functional dependencies considered. *S*_i_ is the metric that integrates contributions from both serotonin and histamine as opposed to the contributions from serotonin or histamine in isolation towards behavior. Compared to existing behavioral grading of depression, *S*_i_, that basis its grading on chemistry, represents a significant advance in stratifying animals.

We independently varied the parameters associated with serotonin and histamine in the model (Fig. 3) and observed their effect on *S*_i_ (Additional file 1: Figure S3). During stress, we found that decreasing *τ*_HA_, corresponding to increased histamine reuptake and increasing *I*_5HT_, corresponding to a surge in extracellular serotonin leads to a significant reduction in *S*_i_ as observed through the large changes from basal levels (Fig. 5). Thus, *τ*_HA_ and *I*_5HT_ maximally contribute to reducing *S*_i_.

In both control and stress conditions, the model predicts that ESCIT administration will cause an increase in *S*_i_ (via reduction in serotonin, counter to the idea that SSRIs increase serotonin), precisely because of ESCIT’s effect on histamine (as above, small changes in histamine create large changes in serotonin). Theoretically SSRIs should increase serotonin levels by inhibiting the serotonin transporter; indeed, we have found this to be the case for acute escitalopram (ESCIT) administration to mice [77]. The model contradicts theory and indeed experiments [46]. This is because the model does not take into account the differing affinities of ESCIT for inhibiting serotonin and histamine reuptake. The much higher affinity of ESCIT for SERTs than OCTs explains the model’s mismatch with experimental data. Nonetheless the model shines an important spotlight on histamine as a means to modulate serotonin. For the next question, we took into account into the varying affinities of ESCIT for serotonin and histamine transporters and asked the model how best to restore stress-induced decreased serotonin to pre-stress control levels. The model predicted that a dual approach, targeting both serotonin and histamine would be the most effective. When we performed the equivalent experiments by coadministering SSRI and a histamine synthesis inhibitor, we almost entirely replicated the model’s predictions experimentally. We have, as yet to find a chronic drug treatment paradigm that does not cause inflammation-induced histamine, thus we have not yet tested the effects of chronic FMH administration. Nonetheless this acute experiment highlights the potential importance of histamine.

## Conclusions

In this work, we combined cutting edge experimental and mathematical tools to study the nexus of the serotonin-driven monoamine theory and the neuroinflammation-driven cytokine theory of depression in a chronic stress model by focusing on serotonin and histamine. We created models to stratify mice that had undergone a chronic mild stress model along a scale, *S*_i_. When *S*_i_ was high our model predicted that a simultaneous increase in serotonin and decrease in histamine would be the most effective chemical strategy to return serotonin to pre-stress levels. We experimentally performed this idea via acute pharmacology and our experiments were almost perfectly in line with the model’s predictions.

In sum, our work reveals that a co-modulatory relationship between serotonin and histamine marks chronic stress in mice. While it is clear that stress and depression pathology extend beyond histamine and serotonin, we suggest that it is not yet time to give up on serotonin. Rather we propose that in vivo serotonin and histamine co-modulatory dynamics be considered as biomarkers in future investigations of the pathology and treatment of depression.

## Supplementary Information

**Additional file 1:** Contains supplementary figures and tables.

## Data Availability

The data sets used and/or analyzed during the current study are available from the corresponding author on reasonable request.

## Abbreviations

5HT: Serotonin
ANCOVA: Analysis of covariance
CFMs: Carbon fiber microelectrodes
CMS: Chronic mild stress paradigm
CV: Cyclic voltammogram
ESCIT: Escitalopram
EZM: Elevated zero maze
FSCAV: Fast-scan controlled-adsorption voltammetry
FSCV: Fast-scan cyclic voltammetry
FMH: Fluoromethylhistidine
FST: Forced swim test
HA: Histamine
*I*_5HT_: Tonic supply of serotonin
*I*_HA_: Tonic supply of histamine
i.p.: Intraperitoneal injection
IT: Current vs. time
OCTs: Organic cation transporters
SEM: Standard error of the mean
SERTs: Serotonin transporters
*S*_i_: Stress index
SPT: Sucrose preference test
SSRIs: Selective serotonin reuptake inhibitors
*τ*_5HT_: Decay/reuptake time constant for serotonin
*τ*_HA_: Decay/reuptake time constant for histamine
TST: Tail suspension test
