# Supplementary material for: A tale of two transmitters: serotonin and histamine as in vivo biomarkers of chronic stress in mice
Source: J Neuroinflammation. 2022 Jun 27;19:167. doi: 10.1186/s12974-022-02508-9 (PMC9235270; doi:10.1186/s12974-022-02508-9)
Supplement: Supplementary file 1 — Additional file 1: Contains supplementary figures and tables. [file 12974_2022_2508_MOESM1_ESM.docx]

# Supplementary Information


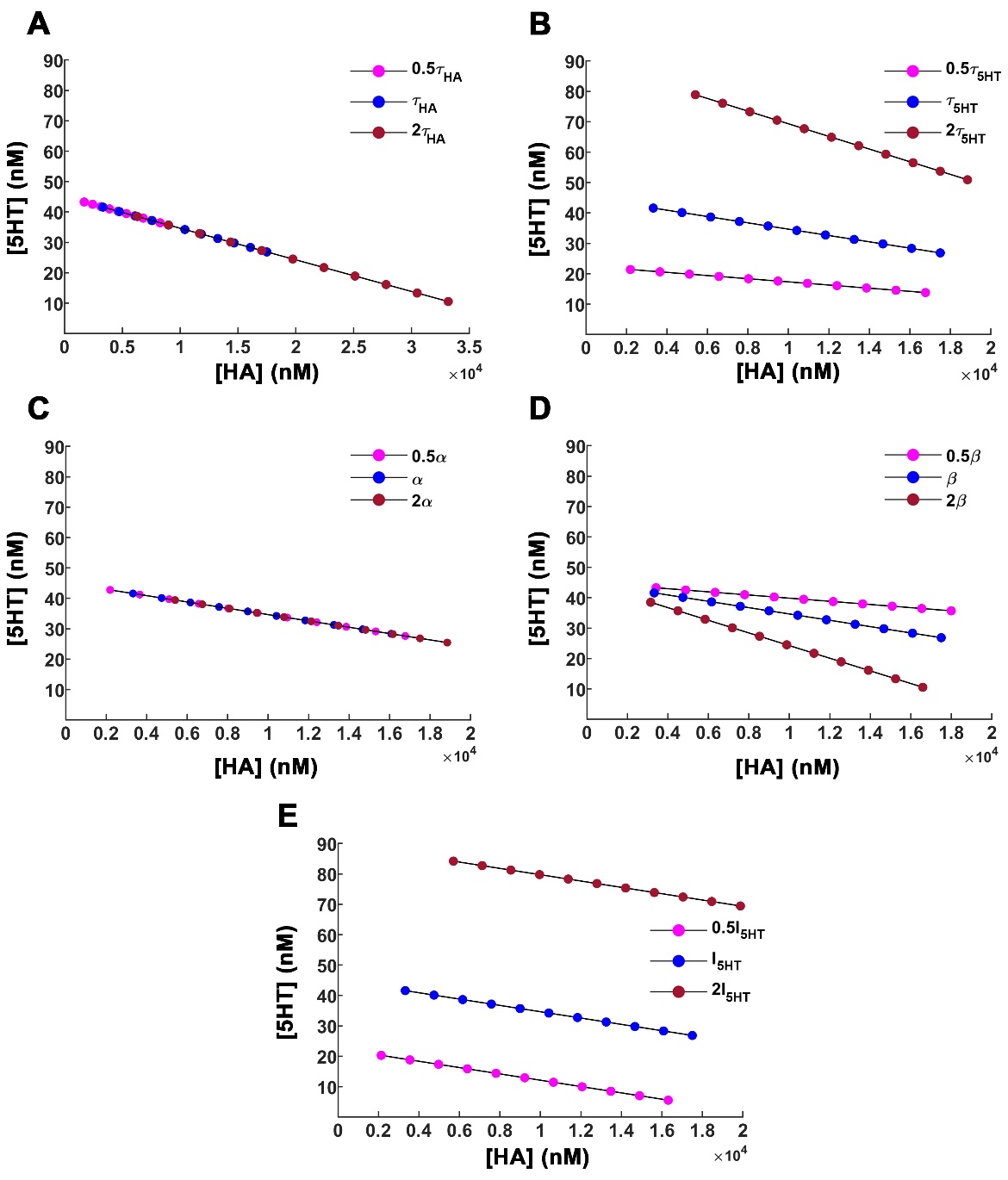


**Figure S1: Effect of parameter variation on the equilibrium states of the system:** The impact of parameter variation on the system’s state is studied for different levels of I_HA_. Each solid circle refers to steady-state histamine and serotonin concentrations. Global sensitivity analysis is performed by providing a two-fold increase and decrease in the parameter of interest, while keeping rest of the parameters fixed. (**A**) τ_HA_ (**B**) τ_5HT_ (**C**) α (**D**) β (**E**) I_5HT_. The blue solid circles correspond to the system's steady states under the basal value of the parameter of interest. Dark red (magenta) solid circles correspond to two-fold increase (decrease) in the parameter. The basal values of the hypothalamic parameters used here are listed in **Table S1**.


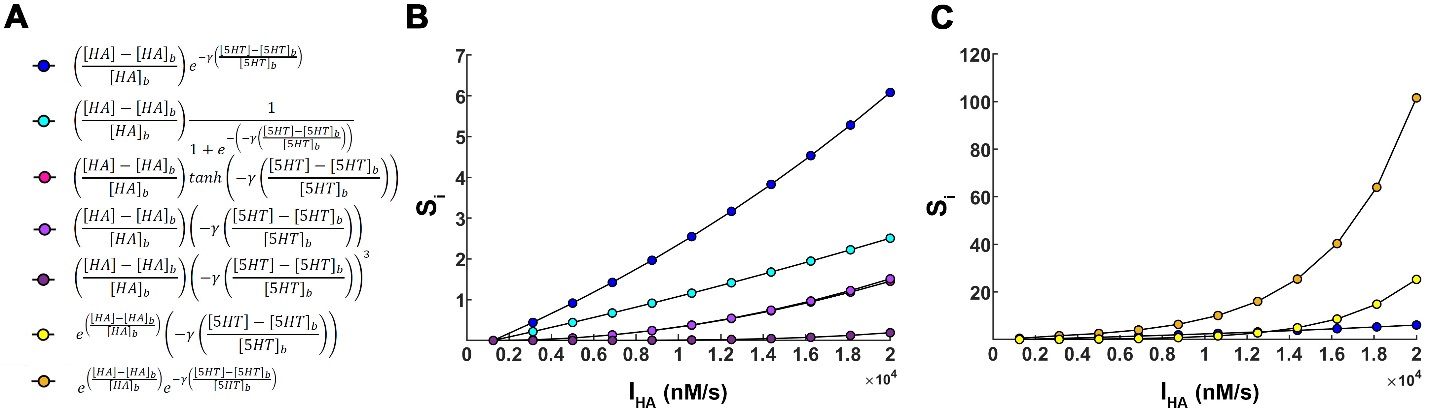


**Figure S2: Different functional forms considered for the Stress Index (S_i_):** (**A**) The different color schemes denote different functional forms for computing S_i_ based on histamine and serotonin steady state concentrations. Here, HA and 5HT refer to the hypothalamic steady state extracellular histamine and serotonin concentrations whereas HA_b_ and 5HT_b_ are the basal extracellular histamine and serotonin concentrations. γ is the strength of serotonin-histamine co-operativity/antagonism. (**B-C**) The functional form considered for S_i_ in the paper is shown in dark blue solid circles. (**B**) Different functional dependencies for S_i_ are plotted as a function of I_HA_ (**C**) Dark orange solid circles demonstrate the case when histamine and serotonin both have an exponential contribution towards S_i_. The yellow solid circle is the case when histamine has an exponential dependence and serotonin has a linear contribution to S_i_. The latter case demonstrates that even when the functional dependencies of histamine and serotonin are interchanged, S_i_ is qualitatively unchanged. The basal values of the hypothalamic parameters used here are listed in **Table S1**.


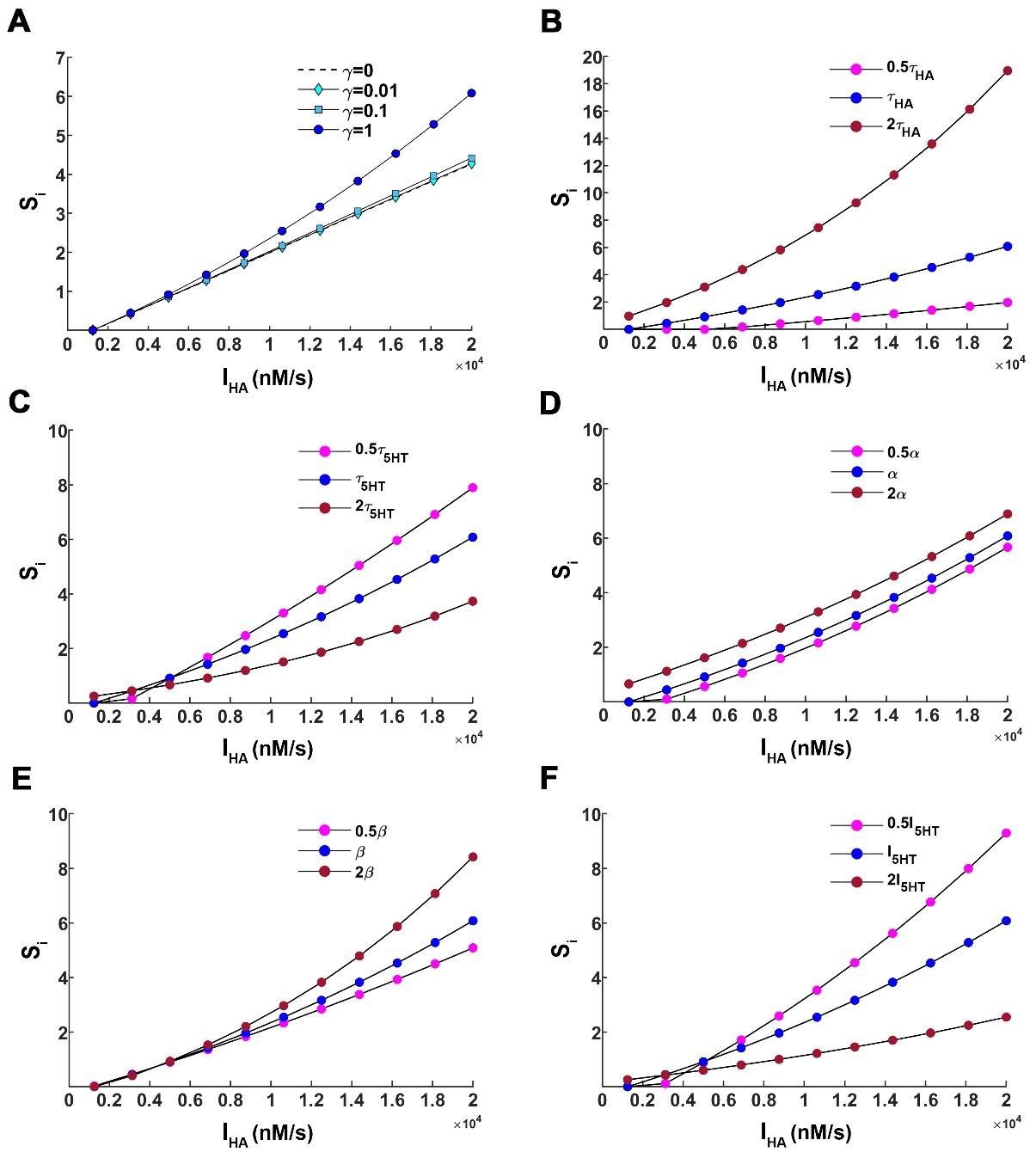


**Figure S3: Impact of parameter variation on Stress index (S_i_) in hypothalamus.** (**A**) The effect of the coefficient of cooperativity/antagonism of serotonin-histamine towards depression, γ, on the Stress Index (S_i_) is illustrated. The dash linear line corresponds to the case with exclusive histamine contribution to S_i_ (γ = 0). An increase in γ builds up the non-linear contribution of serotonin towards S_i_ for increasing I_HA_. (**B-F**) Global sensitivity analysis is performed by providing a two-fold increase and decrease in the parameter of interest while keeping rest of the parameters fixed. (**B**) τ_HA_ (**C**) τ_5HT_ (**D**) α (**E**) β (**F**) I_5HT_. The blue solid circles depict the system's steady-state under the basal value of the parameter of interest. Dark red (magenta) solid refers to a two-fold increase (decrease) in the parameter. The basal values of the hypothalamic parameters used here are listed in **Table S1**.


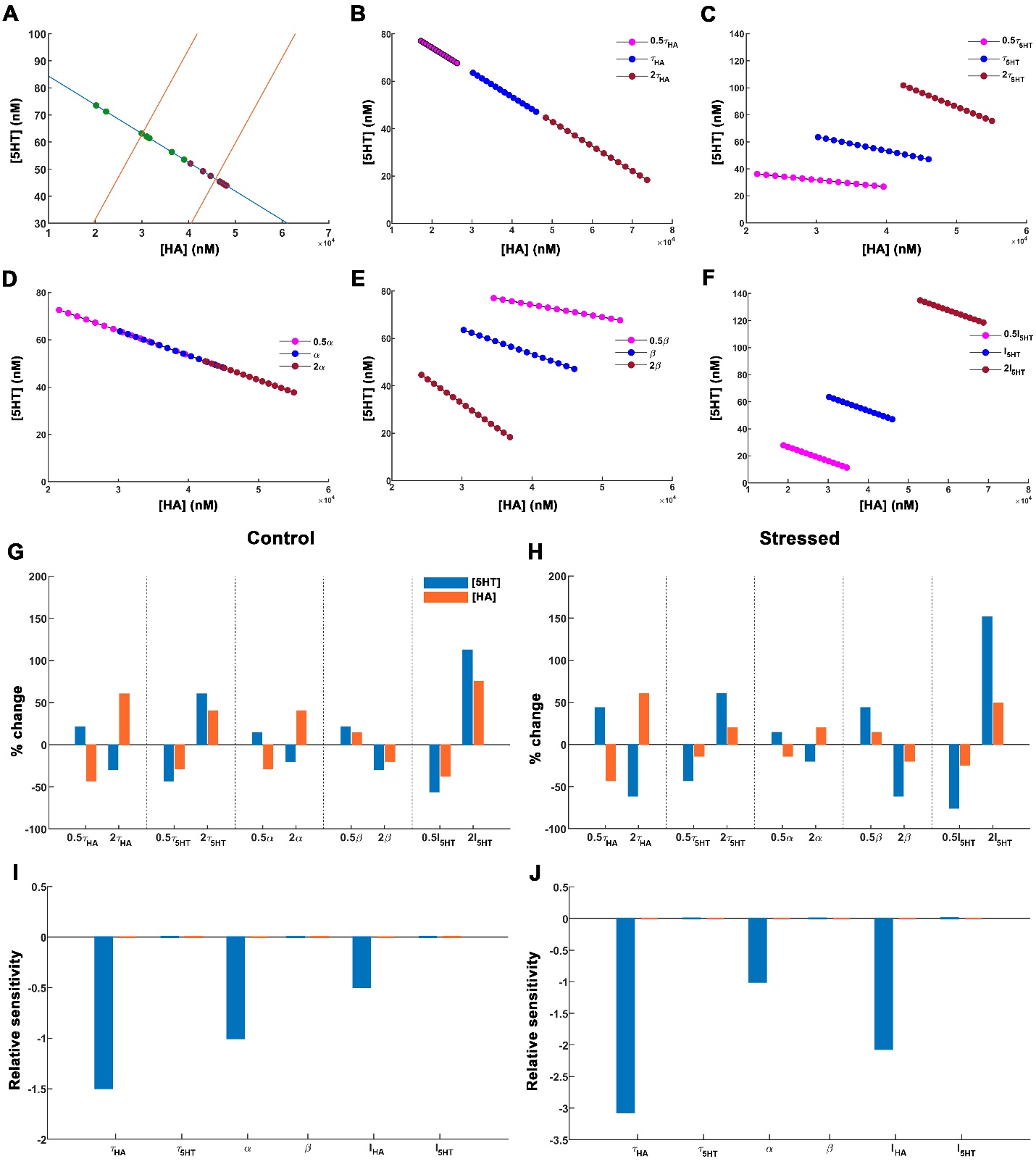


**Figure S4: Parameter sensitivity analyses of the system in hippocampus.**(**A**) The green (red) solid circles are data points with basal hippocampal serotonin levels and associated basal histamine levels, obtained by extrapolating the hypothalamic stress-induced depression model to hippocampal serotonin data, of healthy control (depressed) mice. Nullcline plots for serotonin (in blue) and histamine (in orange) are obtained for two values of I_HA_ – normal physiological state associated with basal value of I_HA_ and, stressed state associated with a high value of I_HA_ = 3.88*10^4^ nMs^-1^ (**B-F**) The impact of parameter variation on the system’s state for different levels of I_HA_. Each solid circle refers to steady-state histamine and serotonin concentrations. Global sensitivity analysis is performed by providing a two-fold increase and decrease in the parameter of interest, while keeping rest of the parameters fixed. (**B**) τ_HA_ (**C**) τ_5HT_ (**D**) α (**E**) β (**F**) I_5HT_. The blue solid circles correspond to the system's steady states under the basal value of the parameter of interest. Dark red (magenta) solid circles correspond to two-fold increase (decrease) in the parameter. (**G-H**) Percentage change obtained in the system’s state under parameter variation is shown with respect to the system’s state at basal values of the respective parameters. The serotonin levels are shown in blue bars and the associated histamine levels in orange bars. (**I-J**) Local sensitivity analysis has been performed to identify the parameters towards which the system is most sensitive and vulnerable. The basal values of the hippocampal parameters used here are listed in **Table S1**.


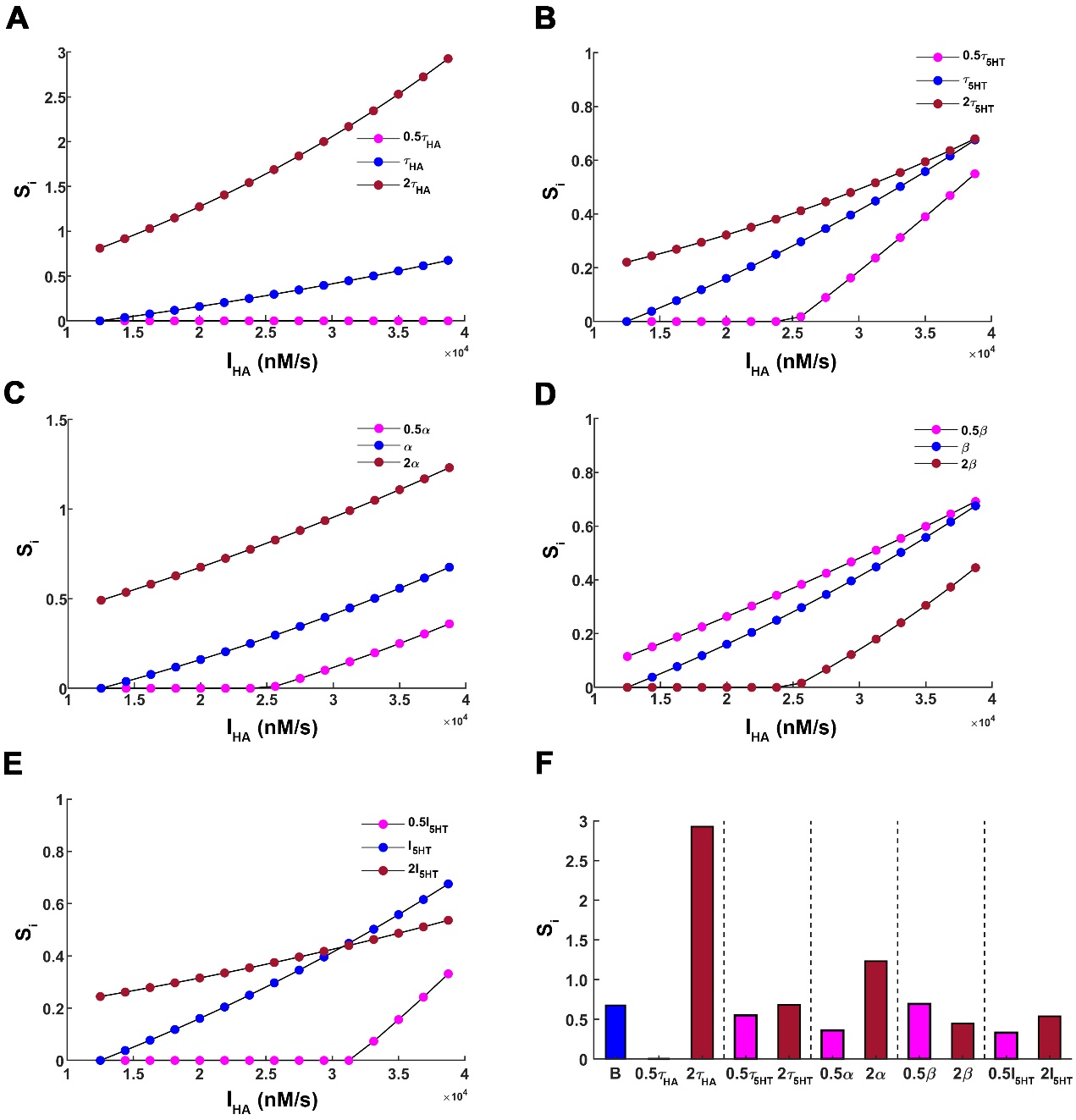


**Figure S5:** **Effect of parameter variation on Stress index (S_i_) in hippocampus.**  S_i_ is a positive scalar function that grades stress-induced depression based on equilibrium hippocampal serotonin and associated hippocampal histamine levels of the state of the system. Global sensitivity analysis is performed by providing a two-fold increase and decrease in the parameter of interest while keeping rest of the parameters fixed. (**A**) τ_HA_ (**B**) τ_5HT_ (**C**) α (**D**) β (**E**) I_5HT_. The blue solid circles depict the system's steady state under the basal value of the parameter of interest. Dark red (magenta) solid refers to a two-fold increase (decrease) in the parameter. (**F**) The S_i_ values for the stressed state (I_HA_ = 3.88*10^4^ nMs^-1^) are shown for different levels of parameters of the system. The blue bar refers to S_i_ for the basal condition i.e. when all the parameters are set to their basal value. The basal values of the hippocampal parameters used here are listed in **Table S1**.


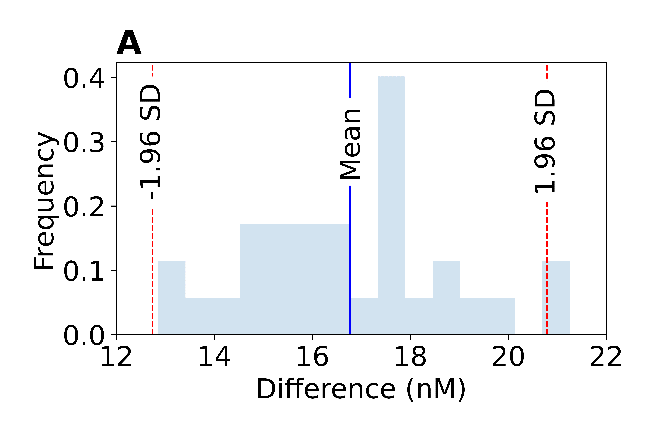

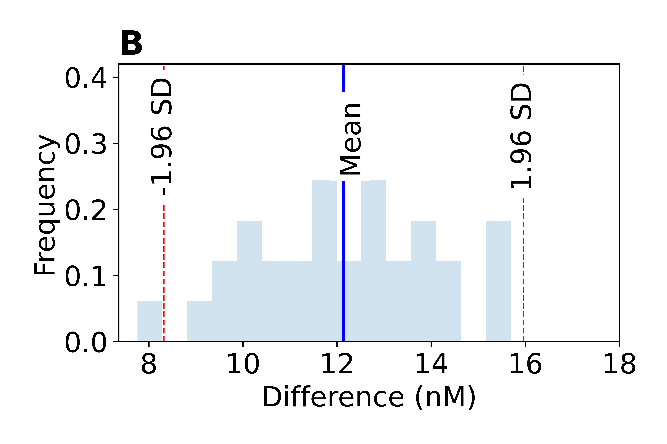

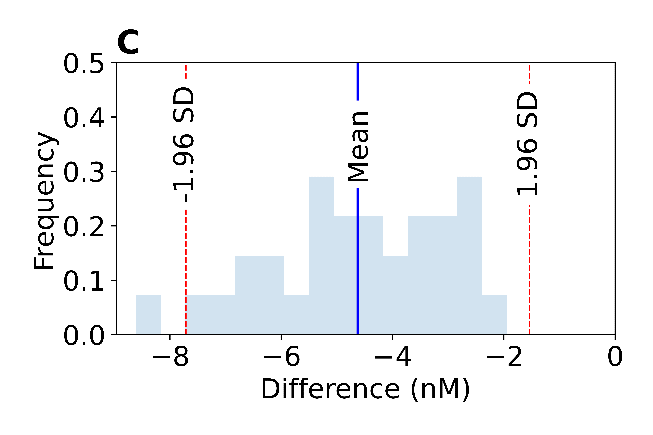

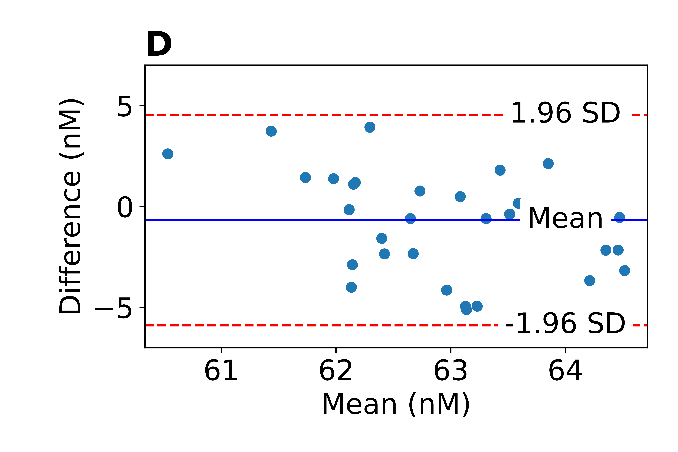

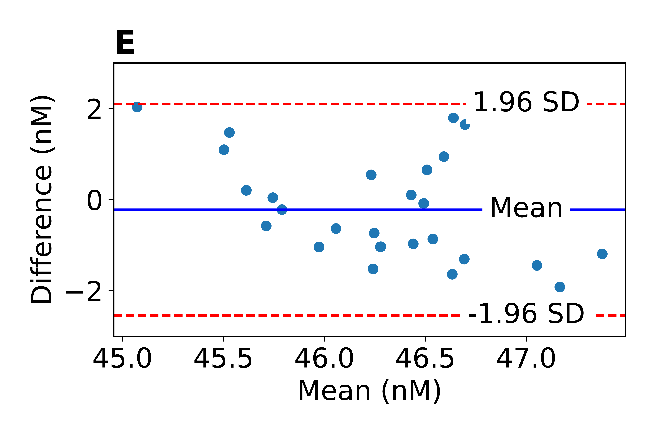

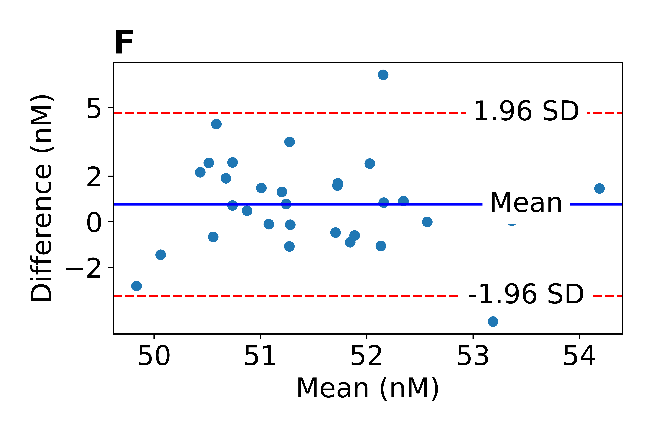


**Figure S6:** **Histograms and Bland-Altman Plots of Baseline Serotonin FSCAV Measurements.** Representation of the frequency of occurrence of the difference between pairs of samples for the first 30 data points: **(A)** Control and CMS mice (16.76 ± 0.38 nM), (**B)** Control and CMS mice (12.14 ± 0.35 nM), and **(C)** CMS and CMS mice (-4.63 ± 0.29 nM). Differences between serotonin baseline samples for control and CMS mice were significantly higher than differences between two groups of CMS mice (**Figure 7B**). Bland-Altman plots to assess systematic bias before and after saline injection for: **(D)** Control mice prior to ESCIT administration (**E**) CMS mice prior to ESCIT administration and (**F**) CMS mice prior to ESCIT and FMH administration. No systematic bias or proportional error are appreciated. The blue lines represent the means of the differences, and the red dashed lines the 95% confidence intervals.


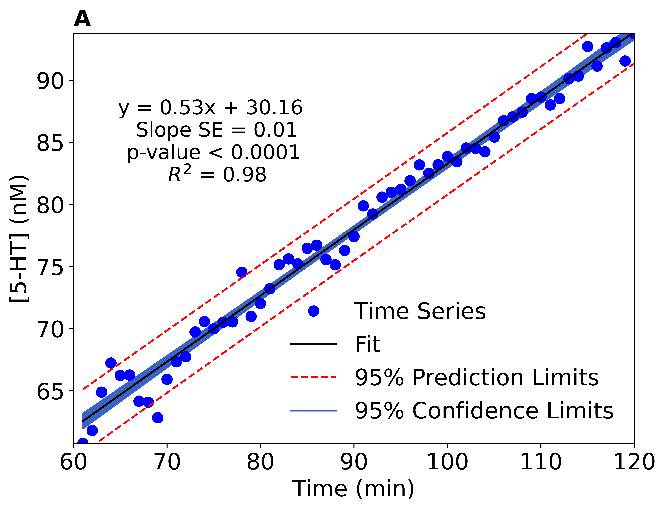

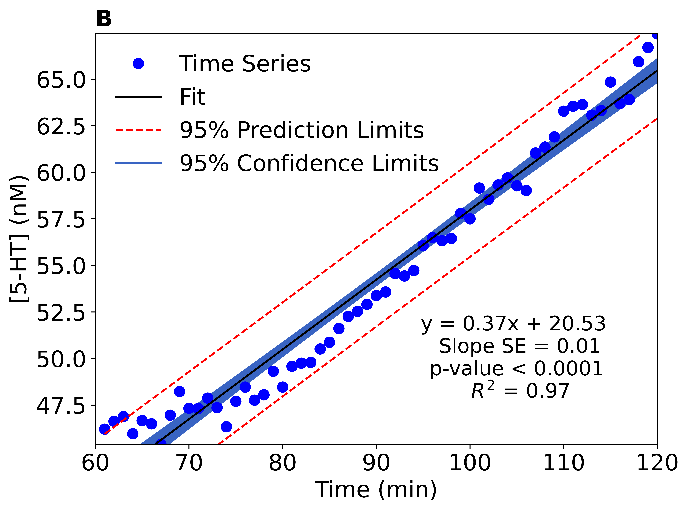

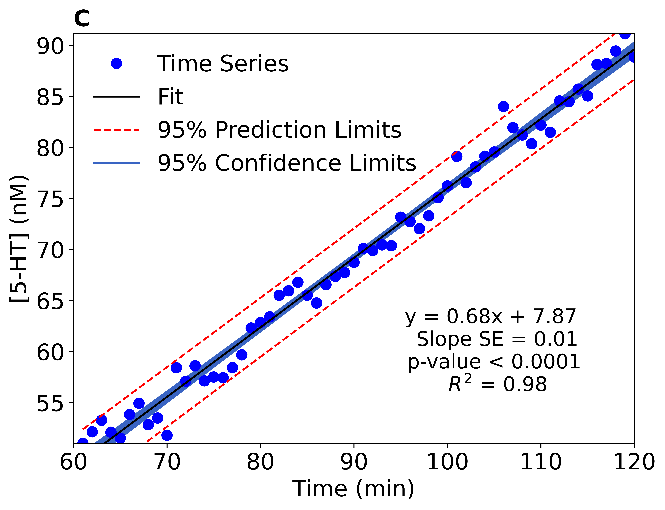


**Figure S7:** **Regression Analysis of Time Series After Drug Administration.** Representation of the experimental serotonin basal concentration for: (**A**) Control mice after ESCIT administration (60 - 120 min), (**B**) CMS mice after ESCIT administration (60 - 120 min) and (**C**) CMS mice after ESCIT + FMH administration (60 - 120 min). A regression line is fitted to each time series. The 95% prediction limits and confidence limits are also shown. The p-values expressed in the plot are obtained from testing the significance of the regression slope.


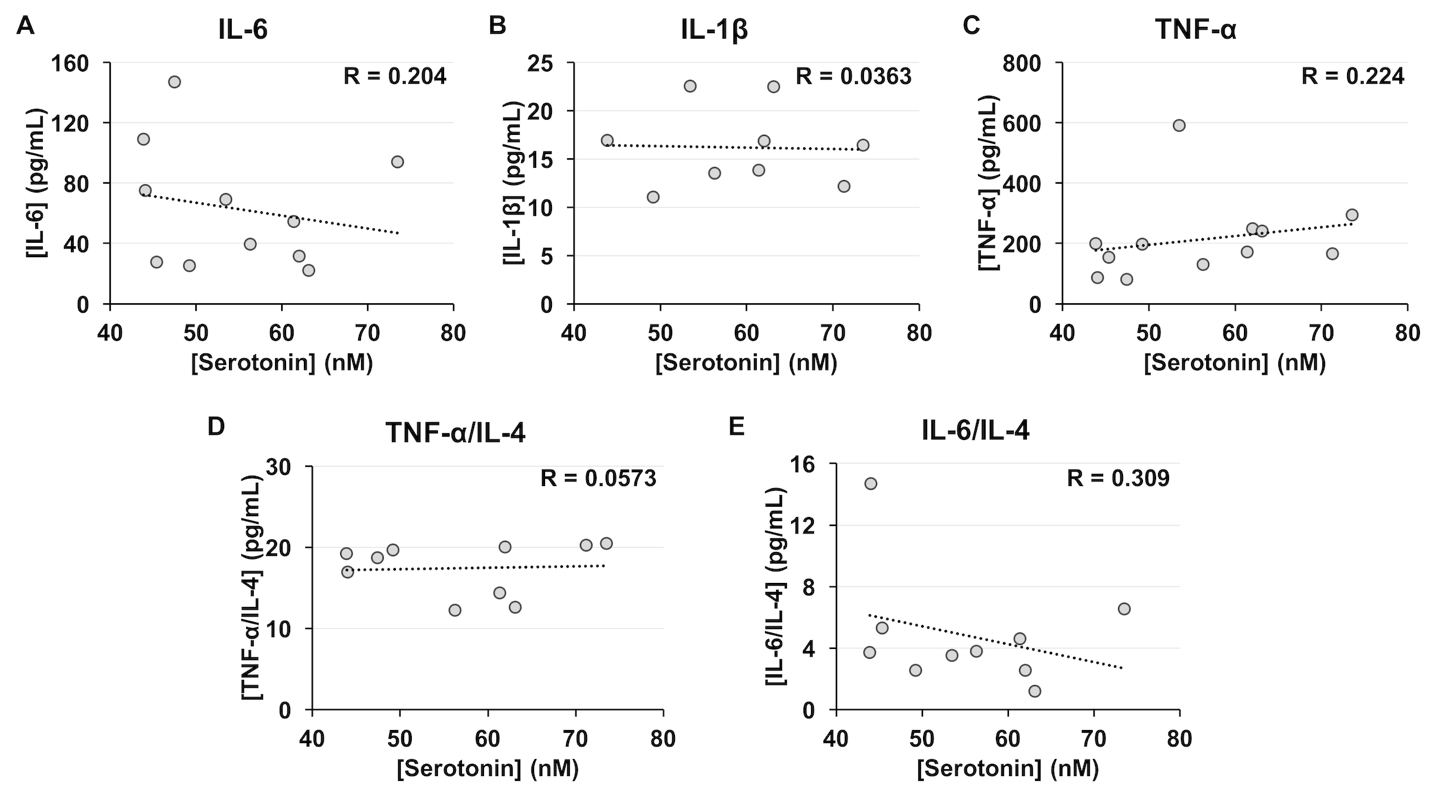


**Figure S8: Cytokine and hippocampal serotonin correlations.** Regressions are shown for (**A**) IL-6, (**B**) IL-1β, (**C**) TNF-α, and the ratios (**D**) TNF-α/IL-4 and (**E**) IL-6/IL-4 compared to extracellular hippocampal serotonin values determined using FSCAV for combined control and CMS mice.

**Table S1:** List of parameters and their values for the hypothalamus and hippocampal model. The parameters for the hypothalamus model have been obtained by fitting experimental data obtained from Samaranayake et al. 2016. Further, for the hippocampal model the serotonergic related parameters are obtained by fitting ambient serotonin levels for depressed and control mice. The hippocampal histamine associated parameters have been obtained by extrapolating the hypothalamic model fit as simultaneous measurement of ambient histamine and serotonin is not experimentally possible yet in the hippocampus.

| **Parameters** | **Hypothalamus model** | **Hippocampus model** | **Description** |
| --- | --- | --- | --- |
| **τ_HA_** | 0.8 s | 0.8 s | Reuptake/ decay time constants of histamine |
| **τ_5HT_** | 0.8 s | 0.8 s | Reuptake/decay time constants of serotonin |
| **α** | 69.7971 s^-1^ | 398 s^-1^ | Rate of increase in histamine levels due to serotonin |
| **β** | 0.0013 s^-1^ | 0.0013 s^-1^ | Rate of decrease in serotonin levels due to histamine |
| **I_HA_** | 1250 nMs^-1^ | 12500 nMs^-1^ | Tonic supply of histamine |
| **I_5HT_** | 56.3250 nMs^-1^ | 118.75 nMs^-1^ | Tonic supply of serotonin |
| **∆I_HA_** | 1875*[0:10] nMs^-1^ | 1875*[0:14] nMs^-1^ | Stress-induced increase in histamine |
| **γ** | 1 | 1 | Strength of cooperativity/antagonism by serotonin |

**Table S2:** List of names of groups of the analysis of covariance, their regression slope means and indexes.

| **Group** | **Time series (min)** | **Slope mean**  **(nM · min^-1^)** | **Number** |
| --- | --- | --- | --- |
| Control baseline | 0 - 30 | -0.02 | 1 |
| Control saline | 30 - 60 | -0.03 | 2 |
| Control ESCIT | 60 - 120 | 0.53 | 3 |
| CMS baseline 1 | 0 - 30 | 0.06 | 4 |
| CMS saline 1 | 30 - 60 | -0.03 | 5 |
| CMS ESCIT | 60 - 120 | 0.37 | 6 |
| CMS baseline 2 | 0 - 30 | -0.01 | 7 |
| CMS saline 2 | 30 - 60 | -0.02 | 8 |
| CMS ESCIT + FMH | 60 - 120 | 0.514 | 9 |

**Table S3:** Analysis of covariance results for the data represented in **Figure 7B**.

| **Source** | **Degrees of Freedom** | **Sum Squares** | **Mean Squares** | **F Statistic** | **Prob > F** |
| --- | --- | --- | --- | --- | --- |
| Group | 8 | 305710 | 3821.40 | 2264.47 | <.0001 |
| Time | 1 | 11931.70 | 11931.70 | 7070.45 | <.0001 |
| Group · Time | 8 | 4037.30 | 504.70 | 299.05 | <.0001 |
| Error | 342 | 577.10 | 1.70 |  |  |

**Table S4:** Tukey *post hoc* test across ANCOVA groups. Probabilities in bold text are of interest.

| **Tukey *post hoc tests for slopes of ANCOVA***  **Pr > \|t\| for H_0_: Slope mean (i) = Slope mean (j)** | | | | | | | | | |
| --- | --- | --- | --- | --- | --- | --- | --- | --- | --- |
| **i/j** | **1** | **2** | **3** | **4** | **5** | **6** | **7** | **8** | **9** |
| **1** |  | 1.0000 | <.0001 | 0.4831 | 1.0000 | <.0001 | 1.0000 | 0.9995 | <.0001 |
| **2** | 1.0000 |  | <.0001 | 0.4654 | 1.0000 | <.0001 | 1.0000 | 0.9996 | <.0001 |
| **3** | <.0001 | <.0001 |  | <.0001 | <.0001 | <.0001 | <.0001 | <.0001 | <.0001 |
| **4** | 0.4831 | 0.4654 | <.0001 |  | 0.3636 | <.0001 | 0.7048 | 0.1452 | <.0001 |
| **5** | 1.0000 | 1.0000 | <.0001 | 0.3636 |  | <.0001 | 0.9999 | 1.0000 | <.0001 |
| **6** | <.0001 | <.0001 | **<.0001** | <.0001 | <.0001 |  | <.0001 | <.0001 | <.0001 |
| **7** | 1.0000 | 1.0000 | <.0001 | 0.7048 | 0.9999 | <.0001 |  | 0.9906 | <.0001 |
| **8** | 0.9995 | 0.9996 | <.0001 | 0.1452 | 1.0000 | <.0001 | 0.9906 |  | <.0001 |
| **9** | <.0001 | <.0001 | **<.0001** | <.0001 | <.0001 | **<.0001** | <.0001 | <.0001 |  |

**Table S5**: Behavioral statistical test results. Each of the behavioral tests was tested for significance using a two-way ANOVA with factors of sex (male and female) and type of mouse (control and CMS), followed by a Tukey-Kramer *post hoc* multiple comparisons. Mean ± SEM and p-values of the multiple comparisons are given in the table. Individual comparisons found to be significant (p < 0.05) are in bold type. Full content of ANOVA tables and all multiple comparisons are available upon request.

| **Behavior** | **Female Control** | **Male Control** | **Control Total** | **Female CMS** | **Male CMS** | **CMS Total** | **p-val:**  **Females** | **p-val: Males** | **p-val:**  **Total** |
| --- | --- | --- | --- | --- | --- | --- | --- | --- | --- |
| Sucrose Preference Test (SPT) | | | | | | | | | |
| 1 hr sucrose preference | 72.5 ± 2.7 | 73.8 ± 4.4 | 73.2 ± 2.6 | 74.7 ± 1.8 | 67.3 ± 3.6 | 71.0 ± 2.0 | 0.9665 | 0.5030 | 0.5102 |
| 3 hr sucrose preference | 73.1 ± 1.8 | 76.3 ± 2.1 | 74.7 ± 1.4 | 68.7 ± 1.5 | 69.4 ± 2.5 | 69.1 ± 1.4 | 0.4201 | **0.0089** | **0.0070** |
| 12 hr sucrose preference | 77.7 ± 1.6 | 88.3 ± 0.9 | 83.0 ± 1.3 | 77.1 ± 1.7 | 83.3 ± 1.4 | 80.1 ± 1.2 | 0.9910 | **0.0087** | 0.0598 |
| Elevated Zero Maze (EZM) | | | | | | | | | |
| Latency to enter open spaces (s) | 38.4 ± 7.3 | 34.8 ± 6.8 | 36.6 ± 4.9 | 52.6 ± 7.6 | 41.3 ± 7.5 | 47.1 ± 5.3 | 0.5144 | 0.9227 | 0.1603 |
| # of entries into closed area | 3.2 ± 0.5 | 3.2 ± 0.6 | 3.2 ± 0.4 | 2.9 ± 0.5 | 1.7 ± 0.3 | 2.3 ± 0.3 | 0.9545 | **0.0389** | 0.0595 |
| Head dips | 25.3 ± 2.0 | 23.1 ± 1.9 | 24.2 ± 1.4 | 21.6 ± 1.6 | 16.9 ± 1.0 | 19.3 ± 1.0 | 0.4256 | **0.0413** | **0.0041** |
| Time spent in closed area (s) | 227.8 ± 5.8 | 232.4 ± 5.0 | 230.1 ± 3.8 | 248.2 ± 4.5 | 251.6 ± 3.2 | 249.8 ± 2.8 | **0.0163** | **0.0280** | **< 0.0001** |
| Forced Swim Test (FST) | | | | | | | | | |
| Pretest + test immobility | 36.7 ± 2.3 | 30.6 ± 6.3 | 34.7 ± 3.2 | 48.7 ± 3.2 | 55.1 ± 10.5 | 52.2 ± 4.4 | **0.0265** | **0.0341** | **0.0037** |
| Test immobility | 50.3 ± 3.7 | 41.3 ± 8.0 | 47.3 ± 4.2 | 61.4 ± 5.5 | 66.1 ± 9.1 | 64.0 ± 4.3 | 0.5964 | **0.0413** | **0.0079** |
| Swimming during test | 35.2 ± 2.3 | 40.7 ± 7.0 | 37.4 ± 3.4 | 27.7 ± 4.6 | 21.7 ± 6.8 | 24.5 ± 3.6 | 0.7264 | **0.0413** | **0.0152** |
| Climbing during test | 7.7 ± 3.1 | 6.8 ± 2.2 | 5.2 ± 1.6 | 3.6 ± 1.5 | 5.7 ± 3.1 | 4.7 ± 1.4 | 0.6768 | 0.9938 | 0.3225 |
| Tail Suspension Test (TST) | | | | | | | | | |
| Pretest + test immobility | 136.0 ± 7.2 | 119.5 ± 7.5 | 128.0 ± 5.3 | 145.7 ± 7.3 | 135.4 ± 5.9 | 140.5 ± 4.7 | 0.7579 | 0.3920 | 0.0726 |
| Test immobility | 68.9 ± 6.1 | 62.2 ± 5.7 | 65.6 ± 4.2 | 74.8 ± 5.8 | 66.2 ± 4.1 | 70.5 ± 3.6 | 0.8721 | 0.9558 | 0.3720 |

**Table S6:** List of names of groups and indexes of the analysis of variance for ratio TNF-α / IL-4.

| **Group** | | **Number** |
| --- | --- | --- |
| **Effect 1** | **Effect 2** |  |
| Control | Male | 1 |
| CMS | Male | 2 |
| Control | Female | 3 |
| CMS | Female | 4 |

**Table S7:** Analysis of variance results for the TNF-α / IL-4 measured ratios.

| **Source** | **Sum sq.** | **d.f.** | **Mean sq.** | **F statistic** | **Prob > F** |
| --- | --- | --- | --- | --- | --- |
| Model | 965.0871 | 1 | 965.0871 | 12.1805 | 0.0009 |
| Treatment | 49.8279 | 1 | 49.8279 | 0.6289 | 0.4306 |
| Interference | 58.4522 | 1 | 58.4522 | 0.7377 | 0.3935 |
| Error | 5.1500e+03 | 65 | 79.2321 | - | - |
| Total | 6.3037e+03 | 68 | - | - | - |

**Table S8:** Tukey-Kramer post-hoc multiple tests results for the TNF-α / IL-4 measured ratios. Probabilities in bold text are of interest.

| ***Tukey-Kramer post-hoc* multiple tests**  **Pr > \|t\| for H_0_: Mean TNF-a / IL-4 (i) =**  **Mean TNF-a / IL-4 (j)** | | | | |
| --- | --- | --- | --- | --- |
| **i/j** | **1** | **2** | **3** | **4** |
| **1** | - | 0.2975 | 0.6675 | 0.2563 |
| **2** | 0.2975 | - | 0.0143 | 1.000 |
| **3** | 0.6675 | 0.0143 | - | 0.0096 |
| **4** | 0.2563 | 1.000 | **0.0096** | - |

**Table S9:** List of names of groups and indexes of the analysis of variance for ratio IL-6 / IL-4.

| **Group** | | **Number** |
| --- | --- | --- |
| **Effect 1** | **Effect 2** |  |
| Control | Male | 1 |
| CMS | Male | 2 |
| Control | Female | 3 |
| CMS | Female | 4 |

**Table S10:** Analysis of variance results for the IL-6 / IL-4 measured ratios.

| **Source** | **Sum sq.** | **d.f.** | **Mean sq.** | **F statistic** | **Prob > F** |
| --- | --- | --- | --- | --- | --- |
| Model | 287.8829 | 1 | 287.8829 | 3.0514 | 0.0854 |
| Treatment | 155.7874 | 1 | 155.7874 | 1.6513 | 0.2033 |
| Interference | 41.6456 | 1 | 41.6456 | 0.4414 | 0.5088 |
| Error | 6.1323e+03 | 65 | 94.3432 | - | - |
| Total | 6.6284e+03 | 68 | - | - | - |

**Table S11:** Tukey-Kramer post-hoc multiple tests results for the IL-6 / IL-4 measured ratios. Probabilities in bold text are of interest.

| ***Tukey-Kramer post-hoc* multiple tests**  **Pr > \|t\| for H_0_: Mean IL-6 / IL-4 (i) =**  **Mean IL-6 / IL-4 (j)** | | | | |
| --- | --- | --- | --- | --- |
| **i/j** | **1** | **2** | **3** | **4** |
| **1** | - | 0.3741 | 0.9736 | 0.9888 |
| **2** | **0.3741** | - | 0.1331 | 0.4936 |
| **3** | 0.9736 | 0.1331 | - | 0.4497 |
| **4** | 0.9888 | 0.4936 | **0.4497** | - |
